# A Review on Natural Fiber Reinforced Polymer Composite for Bullet Proof and Ballistic Applications

**DOI:** 10.3390/polym13040646

**Published:** 2021-02-22

**Authors:** N. M. Nurazzi, M. R. M. Asyraf, A. Khalina, N. Abdullah, H. A. Aisyah, S. Ayu Rafiqah, F. A. Sabaruddin, S. H. Kamarudin, M. N. F. Norrrahim, R. A. Ilyas, S. M. Sapuan

**Affiliations:** 1 Institute of Tropical Forestry and Forest Products (INTROP), Universiti Putra Malaysia (UPM), Serdang 43400, Selangor, Malaysia; a.humaira.aisyah@gmail.com (H.A.A.); ayu.rafiqah@yahoo.com (S.A.R.); atiyah88@gmail.com (F.A.S.); sapuan@upm.edu.my (S.M.S.); 2 Centre for Defence Foundation Studies, Universiti Pertahanan Nasional Malaysia (UPNM), Kem Perdana Sungai Besi 57000, Kuala Lumpur, Malaysia; 3 Department of Aerospace Engineering, Universiti Putra Malaysia, Serdang 43400, Selangor, Malaysia; 4 School of Industrial Technology, Universiti Sains Malaysia, Pulau Pinang 11800, Pulau Pinang, Malaysia; 5 School of Industrial Technology, Faculty of Applied Sciences, Universiti Teknologi MARA (UiTM), Shah Alam 40450, Selangor, Malaysia; sitihasnahkam@uitm.edu.my; 6 Research Center for Chemical Defence, Universiti Pertahanan Nasional Malaysia (UPNM), Kem Perdana Sungai Besi 57000, Kuala Lumpur, Malaysia; faiznorrrahim@gmail.com; 7 School of Chemical and Energy Engineering, Faculty of Engineering, Universiti Teknologi Malaysia (UTM), Skudai 81310, Johor, Malaysia; ahmadilyas@utm.my

**Keywords:** ballistic, biocomposites, bullet proof, natural fiber, polymer composite

## Abstract

Even though natural fiber reinforced polymer composites (NFRPCs) have been widely used in automotive and building industries, there is still a room to promote them to high-level structural applications such as primary structural component specifically for bullet proof and ballistic applications. The promising performance of Kevlar fabrics and aramid had widely implemented in numerous ballistic and bullet proof applications including for bullet proof helmets, vest, and other armor parts provides an acceptable range of protection to soldiers. However, disposal of used Kevlar products would affect the disruption of the ecosystem and pollutes the environment. Replacing the current Kevlar fabric and aramid in the protective equipment with natural fibers with enhanced kinetic energy absorption and dissipation has been significant effort to upgrade the ballistic performance of the composite structure with green and renewable resources. The vast availability, low cost and ease of manufacturing of natural fibers have grasped the attention of researchers around the globe in order to study them in heavy armory equipment and high durable products. The possibility in enhancement of natural fiber’s mechanical properties has led the extension of research studies toward the application of NFRPCs for structural and ballistic applications. Hence, this article established a state-of-the-art review on the influence of utilizing various natural fibers as an alternative material to Kevlar fabric for armor structure system. The article also focuses on the effect of layering and sequencing of natural fiber fabric in the composites to advance the current armor structure system.

## 1. Introduction

In the past few years, consumers’ awareness of new products has been very strict from renewable resources. Green marketing, new directives on recycling, social influence and change perceived value has led consumers to switch to environmentally friendly products. Military confrontations and wars never seem to stop throughout the world history. The level of personal and property protections against the threats in the battlefield and in riot situation has been developed in line with the advancement of the attacking weapons. Thus, different materials were used as body shield and these include animals’ skin, as well as wooden shield and metal shield. Ballistic protective materials are mainly used for personnel protection [1]. The used of ballistic shields are layered composites that are most often described are made up of a hard layer such as ceramics, and soft input. A projectile striking a hard plate can become deformed and cause the fragmentation of a fragile armor, thus posing a risk for users. The function of soft input is capture and absorb the remaining energy of blunt projectiles and projectile fragments damaged first shielding layer [2]. The use composite made up of polymer and natural fiber become extensively used in bullet proof as well as ballistic application.

Natural fibers can be divided into two categories which are animal-based fibers and plant fiber. Cocoon silk, chicken feathers, wool and spiders Silk is usually used as an animal fiber, mainly for biomedical applications, such as implants. These biological products must be biodegradable, which means the ability to break down and absorb the human body or have biocompatibility to avoid harmful to the human body [3]. One of the issues of natural fibers is information dispersion and mechanical differences. Moreover, the producers and users of these materials lack standards about methods of collecting, processing, post-processing natural fibers adds to the complexity of selection. These issues are actually a key deterrent to the widespread use of natural fibers in different fields [4]. Natural fiber polymer composite materials are light in weight with reasonable strength and if engineered effectively, such composites can give more comfort to the wearer in addition to being environment-friendly. Some of the properties that make natural fibers become an attractive alternative to synthetic fibers are low cost, light weight, minimal health hazards during processing, biodegradable, reasonably good specific strength and modulus, good thermal and acoustic insulation characteristics, ease of availability [5]. Composite materials are mainly divided into three categories such as metal composites, ceramic composites and polymers composite materials. Among the three types, polymer composites were received high demand due to their low weight to strength ratio which was applied for many applications such as cars interior, airplanes, spacecraft, ships, civil construction, packaging and sports goods. The use of polymer composites is growing exponentially due to its good mechanical properties, chemical resistance, and corrosion resistance. However, their fire resistance behavior caused serious safety problems in the use of the following purposes polymer composites [6].

In the service life of aerospace, ship, energy transmission and automobile structures, foreign objects may cause ballistic impact load from events such as bird strikes, hail, shrapnel, runways, fragments, bullets and explosive fragments [7,8,9,10]. As well as the potential of penetration, such impacts can lead to extensive delamination, resulting in degradation of the structural performance. Majority of these structures are not intended to serve as armored forces, and because of this possibility that they may be impacted at high speed low-quality fragments, fully understand its response to ballistics requires shock loads and related damage mechanisms is needed [11]. In high performance applications such as aerospace and defence, the primary objective is to reduce the weight of the structure for the intended usage. Effect of target thickness on ballistic impact performance becomes an important consideration for further investigations. The support conditions have imperative influence on the impact response in low velocity impact regime, but the response of the structural element is generally independent of its support conditions in high velocity impact. There are many experimental studies on the ballistic impact of polymer composite materials [12].

Previous research on natural fibers dates back to 2007 when, Wambua et al. [13] investigated and compared the ballistic characteristics of natural fiber reinforced polypropylene (PP) polymer composites (NPP composites) with steel plate backed NPP composites, and the NPP composites with steel plates as facing and backing material. Several studies are available in literature on ballistic impact behaviour of composite structures. The focus of the present study is on analytical methods on ballistic impact behaviour of composites. An analytical model of normal impact and perforation of cylindrical conical projectiles on laminated Kevlar/polyester composites was developed by Rodriguez et al. [14].

**Figure 1:**
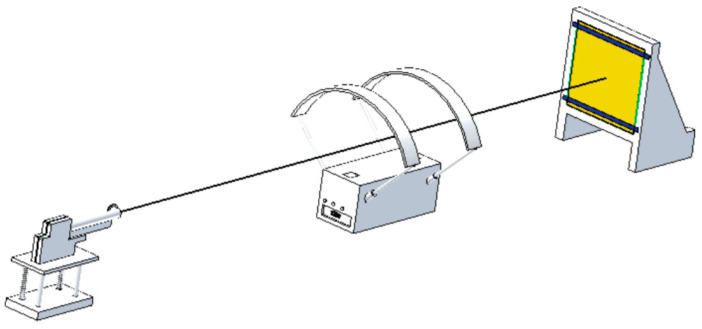
Ballistic test set up. (Adapted with copyright permission from Karahan (Cai, Z., et al. 2016)).

In general, the metal hard armor performance is determined by the material’s properties, as in the front and rear the armor. Hence, the front face erodes the projectile while the laminate of the target rear face absorbs the residual kinetic energy of the projectiles to prevent penetration [15]. Among many existing methods, the most commonly used to determine the ballistic efficiency of the armor are determining the ballistic limit and is measured behind the armor. To test personal protective armors various types of test setups are available like gas gun and powder gun barrel. The setup typically consists of a projectile propelling device (gun), velocity measurement devices, and high speed cameras. Figure 1 shows set up to gauge the applicability of various materials in different types of ballistic conditions.

**Figure 2:**
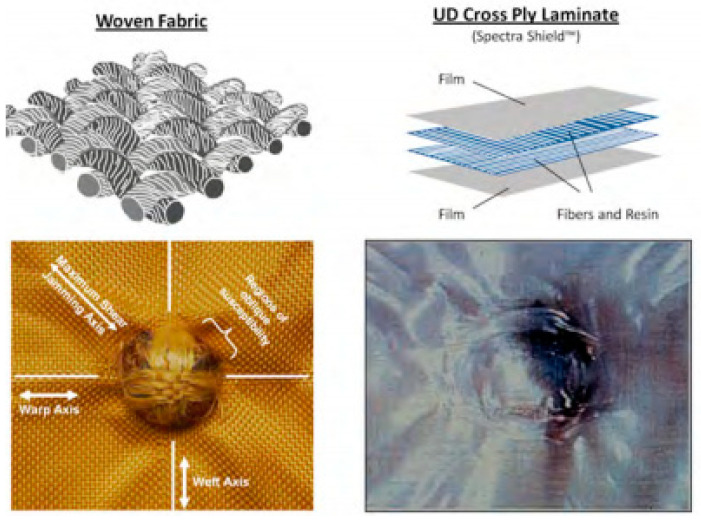
Woven and UD fabric laminate with ballistic impact. (Adapted with copyright permission from Cavallaro et al. [17]).

**Figure 3:**
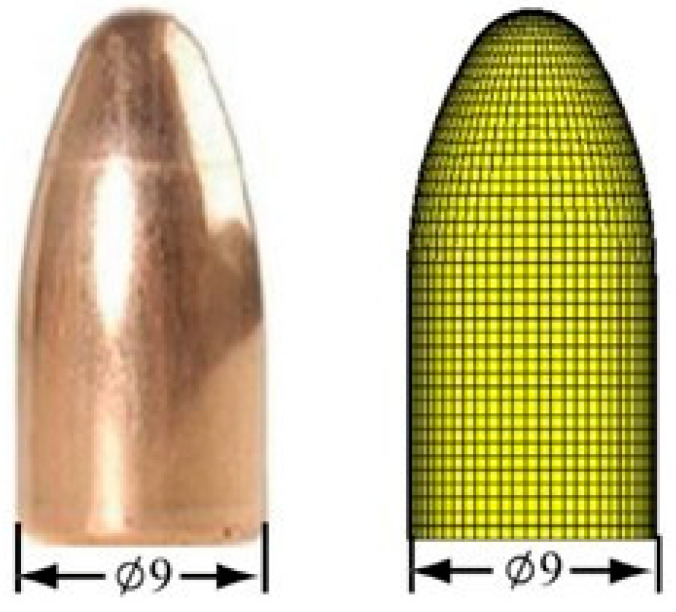
Size of gun bullet. (Adapted with copyright permission from Cai et al. [18]).

Modern body armor can be divided into two categories, which are hard body armor and soft body armor, depending on the type of material used. Generally, the protection level of hard body armor is better than that of soft body armor. Soft body armor with adequate ballistic protection is preferred due to their flexibility, lightweight and comfort properties. Hard body armor is made from rigid materials such as ceramics, reinforced plastics, metal plates and composites. Meanwhile, soft body armor consists of several layers of high performance materials produced from ballistic fibers [16]. Traditionally, soft body armors for ballistic protection were manufactured using layers of woven fabrics stitched together; now they include laminates stacked with nonwoven, unidirectional (UD) layers and combinations of woven or nonwoven laminates. Figure 2 shows the woven and UD fabric laminate with ballistic impact, and Figure 3 shows general actual geometry of gun bullet with its computer modelling.

**Figure 4:**
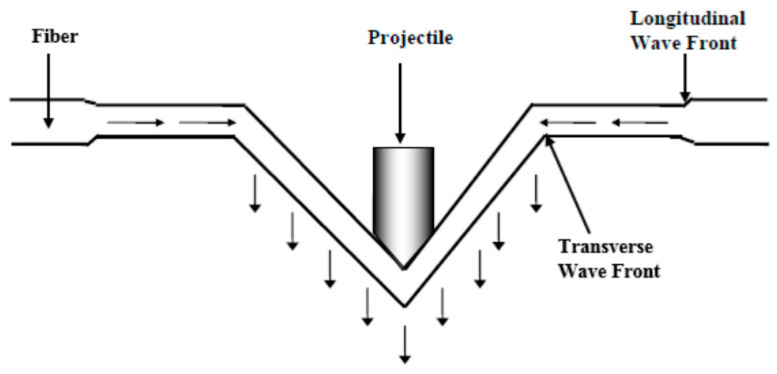
Mechanism of ballistic protection. (Adapted with copyright permission from Cheeseman and Bogetti [19]).

The mechanism, by which ballistic protection is achieved, involves absorption of energy of the projectile or fragment. These projectiles or fragments have large kinetic energy decided by their mass and projected velocity. At the time of striking the target velocity of the projectile or fragment are equally important [19]. Figure 4 shows the mechanism of ballistic protection. Current soft body armors used for ballistic protection are worn to protect the torso and extremity regions. Generally, they are developed in conjunction with rigorous standards and specifications to ensure proper performance and reliability levels against ballistic and fragment threats. For example, the National Institute of Justice (NIJ) prepared the “Ballistic Resistance of Body Armor NIJ Standard-0101.06” to categorize ballistic threats including projectile types, sizes, and velocities; establish deformation limits; develop sample conditioning protocols; and specify acceptance testing procedures for non-military body armors. Table 1 lists the NIJ Standard-0101.06-specified projectile types (deformable, steel-jacketed, and high-hardness core, armor-piercing, etc.), velocities, and maximum allowable back-face signature (BFS) depths [17].

## 2. Classification of Natural Fibers and the Manufacturing Techniques

Natural fibers have excellent properties that are evident in different industries. Natural fibers are currently attracted by their high-quality mechanical properties and biodegradable characteristics, especially in the automotive industry and for general engineering applications. Furthermore, many researchers now work on green materials and focus them on their work [20]. The increase in demand for engineering materials has led to enthusiastic wide-ranging research and the development of new and improved materials, particularly from the polymer composites industry. Natural fibers have mostly been used in the polymer composite industry as a reinforcement for the biocomposite product. Interest in natural fibers growing for a number of reasons, including its comparative advantages of replacing low-cost synthetic fiber reinforced plastics with improved sustainability, eco-friendly and renewable sources. In addition, fiber reinforced materials in structural plastics have been commonly used by the industry for various applications [21,22].

**Figure 5:**
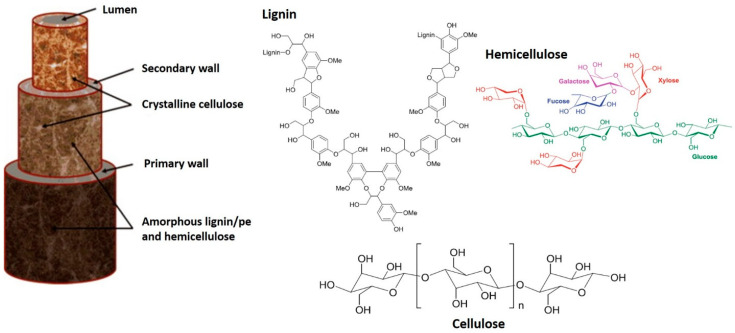
Structure of natural fibers.

Natural fibers has a unique structure which consist of cell wall structure which is divided into three major structural parts [23]. The microfibril angle and arrangement inside the cell wall decide the properties of fibers. Cell wall mainly made up of two cell walls, primary cell wall (S1) and secondary cell wall (S2). Primary cell wall propagates at the time of growth of plant. Secondary cell wall is made up by three layers and each layer carries long chain of microfibril [24]. Hemicelluloses molecules are net-like structure and make bond with cellulosic fibrils. Cellulose and hemicelluloses make network together and lignin and pectin provide an adhesive quality. These adhesive properties are responsible for strength and rigidity of cellulosic fibers. Secondary layer (S2) decides the physical and mechanical strength of fibers. Normally high level of cellulose content and lower microfibrillar angle provide better strength properties [25]. Table 2 shows the comparisons on the physical and mechanical performance of natural fiber with synthetic fiber. Figure 5 shows the schematic diagram structure of natural fiber.

Natural fiber brings in several disadvantages as they are incompatible with polymers when used in raw state such as high water absorption, dead cells, wax, and oil. To overcome this, their surface needs to be modified. The main purpose of surface modification is to increase the properties of natural fibers for it to impart better strength in composites system. Surface modification is done either by chemical treatment, enzymatic treatment, corona or plasma treatment, or by addition of coupling agents [26]. These treatments mostly target the amorphous part of cellulose region hence improve compatibility between fiber and polymer matrix. The amorphous part of cellulose consist of multiple hydroxyl groups which impart a polar nature to the fiber which lead to a poor bonding with polymer matrix. Therefore, surface modification was subjected with the purpose of reducing the polar nature of the fiber by reducing/removing the hydroxyl groups and help to increase the fiber-matrix adhesion and enhance its mechanical properties [27].

**Figure 6:**
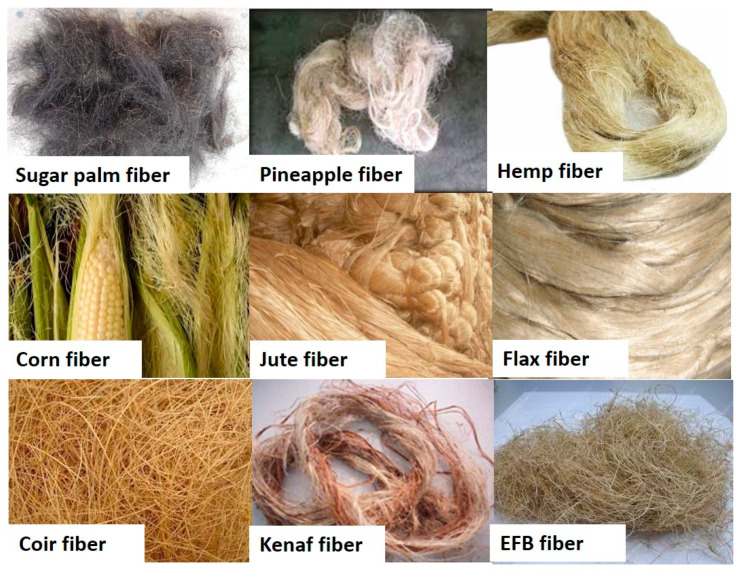
Various types of natural fiber.

Plants that generate natural fibers (Figure 6) are classified as primary and secondary, depending on their use. Primary plants are those which are grown specifically for their fibers, such as cotton, jute, kapok, hemp, kenaf, sisal and secondary plants, which are produced as by-products such as banana, coconut coir, pineapple and oil palm [28]. Near about 30 million tons of natural fibers are produced every year and used as component of many manufacturing processes like clothing, packaging, paper making, automobiles, building materials, and sports equipment. Other than plant fibers, various animal fibers also have different types such as products from the wool, silk, feathers, avian fiber, and animal’s hairs which are prime resource. Natural fibers have been used for a long time in many developing countries [25]. Low cellulose causes weak bonding between adjacent moisture content and fiber causing better interfacial adhesion between the components. Hence, improving the overall suitability to high-end ballistic applications. Table 3 tabulated types of hybrid natural fiber/synthetic fiber reinforced polymer matrix use in ballistic application.

After many years, the production of synthetic fibers has dominated the global industry. Renewed interest in natural reinforcements is remarkable, particularly as a glass fiber substitute in the automotive industry [22]. Synthetic fiber has been replaced by natural fiber for high performance materials such as those in the automotive and aerospace industries. In Germany, the automotive industry intends to manufacture biodegradable and recyclable components that provide incentives for the use of natural fiber. Natural fibers have attracted the attention of researchers and technologists because of the advantages that these fibers offer over synthetic reinforcement materials due to their environmental and economic benefits. The utilization of natural fibers increase the degradation properties of the composites materials thus reduce the environment pollution [45]. It also preferred because of their lower impact towards human health and environment during their use [46]. In addition to this, the energy consumption by natural fibers during their production is only 17% as compared to synthetic fibers like glass fiber [47]. The application of natural fibers also reported able to reduce the value of carbon footprint up 18% which can help to reduce the effect of greenhouse gas emission [48]. Besides, the application of natural fibers particularly coming from local natural resources able to contribute a sustainable regional development, reduction of transport cost and associated environmental impacts. The utilization of local natural sources can help to contribute to the economy and social development especially for rural areas which expected can help on promoting better life quality in ones communities [49].

Natural fibers have been selected for their advantages such as low density, high stiffness, low cost, low-risk manufacturing and renewable resources. Though, it also has certain disadvantages, such as unpredictable consistency depending on unforeseeable conditions such as environment and moisture absorption, lower durability and lower processing temperature. The absorption of moisture due to the hydrophilic properties of natural fibers adversely affects mechanical properties such as flexural strength, flexural modulus and toughness of fractures [22].

Most of the disadvantages identified can be overcome by effectively hybridizing natural fibers with synthetic or natural fiber. The difference in strength is one of the main reasons why natural fibers unable fully replace the glass fibers. However, these drawbacks can overcome by many ways such as fiber modification and fiber treatments. This method was commonly used to enhance the existing properties of fiber especially by using alkali treatment which are convenient and cheap. Studies have shown that alkali treatment with sodium hydroxide (NaOH) solution can improve mechanical strength of the natural fiber [50,51,52,53]. The fibers used for ballistic protection should have low density, high strength and high energy absorption capability. The ballistic performance of a material depends on its ability to absorb energy locally and to distribute it across a whole structure. For textile fibers, the tenacity and elongation at rupture, the sonic velocity (the velocity of sound in textile) of the fibers are important parameters determining the protection that they can provide [54].

Previous researcher investigated and compared the ballistic characteristics of natural fiber reinforced polypropylene composites (NPP composites) with steel plate backed NPP composites and NPP composites with steel plates as facing and backing material. Natural fibers considered for the study were flax, hemp, and jute fibers. Composites were fabricated by compression molding and a fiber volume fraction of 46% was maintained [13]. Yahaya et al. [29,31] explored the potential of kenaf fibers as a substitute for aramid which is commonly used in ballistic armors. Hybrid composites were fabricated from kenaf fibers, aramid fibers, and epoxy as matrix. Stacking sequences were varied and the composites were subjected to tests like quasi static penetration and high velocity impact tests. Damage assessment was also done to study the failure modes as a result of hybridization which revealed failure mechanisms as combination of fiber fracture, delamination, and shearing of fibers at the point of impact [29,55]

Monteiro et al. [56] explored the potential of a relatively unknown, new natural fiber extracted from fique plant in polyester composite backed MBAS. The authors observed improvement in the visco-elastic and thermal properties of polyester resin with the introduction of fique fibers. The depth of indentation in the clay body with fique/polyester composite was 15 mm in comparison to 23 mm with polyester/Kevlar composite. A cost analysis carried out by the researchers showed that polyester/fique composite-based MBAS would be 13 times less expensive than polyester/Kevlar composite [57]. There also a research on hybrid composite studied on ballistic impact performance of hybrid polypropylene based composites reinforced with 2D/3D Kevlar and basalt fiber. Two types of composites were fabricated, one with a symmetric stacking sequence and the other with a non-symmetric stacking sequence. Hybridization was achieved by weaving fabric with basalt and Kevlar yarns. Ballistic impact tests were conducted with a 9 mm full metal jacket projectile of lead core and brass jacket at velocity ranging between 365 m/s and 435 m/s. Series of rounds were fired on both the laminates. For the non-symmetric laminate basalt fabric side was the front face while the back face had 3D Kevlar fabric [58].

## 3. Polymer Composites Manufacturing Techniques

Normally, natural fiber polymer composites are fabricated by using traditional manufacturing techniques which are designed for conventional fiber reinforced polymer composites and thermoplastics. These techniques include hand lay-up, resin transfer molding (RTM), vacuum infusion, compression molding, direct extrusion, compounding and injection molding. Nevertheless, such techniques have been well developed and accumulated experience has proved their ability for producing composites with controllable quality [59]. Table 4 shows the list of various manufacturing technique in ballistic application, and Table 5 shows the advantages and limitations of polymer composites manufacturing techniques.

### 3.1. Hand Lay-Up

Previous research was used, hand lay-up technique which process non-woven kenaf fiber and Kevlar epoxy hybrid in three different configurations. The hybrid was cured at room temperature for 24h at static load [31]. Other researcher developed ballistic resistance composites by using laminating high performance synthetic fibers such as aramid (Kevlar, Twaron) or a combination of two types of fibers.by using hand lay-up techniques [29]. There was also a research using synthetic fiber such as aramid and ultra-high-molecular-weight polyethylene (UHMWPE) lay-up with epoxy and pineapple fiber. The results indicate that a hard armor with a ceramic front followed by the PALF/epoxy composite meets the National Institute of Justice (NIJ) international standard for level III protection [30]. Hybrid composite from sisal fiber and polyaramide fiber was lay-up with epoxy possesses good tensile, flexural and impact strength [32].

### 3.2. Resin Transfer Molding

Resin transfer molding (RTM) was a method that was frequently used in automotive and aerospace sector in manufacturing. However, RTM process also frequently used for composite in ballistic application. In this process, layers of dried fibers are arranged inside and outside of the mold, in order to close it, the resin is injected at a certain pressure to wet the fibers. Previous research was used polyaramid/vinyl ester blends with Kevlar fiber for ballistic application by using RTM method. The mechanical properties was increased as increase in fiber content [34]. Study on woven fabric with unsaturated polyester resin provide high strength due to multilayer and three-dimensional angle interlock woven fabrics arrangement. High velocities impact testing was conducted to analyze the properties for this composite. From the analysis showed three-dimensional angle interlock woven composites absorb more impact energy compare with three-dimensional angle interlock woven fabric [35].

### 3.3. Vacuum Infusion

Vacuum-assisted resin infusion molding technique (VARIM) was used for manufacturing of specimens. Other researcher was used woven glass and graphite fiber reinforced with epoxy and stacking the composite in 28 layers. From the analysis shows hybrid composite possess strong ballistic limit velocity compare with single fiber [33]. Bulut et al. [39] study on composite from carbon-aramid, epoxy and aramid epoxy. These composites were manufactured by vacuum-assisted resin infusion molding technique. The sample set up for hybrid composite was 38 layers and composite sample only 15 layers due to carbon-aramid fiber is thinner than aramid. From the impact velocity test, carbon-aramid/epoxy composite shows high performance and the material was chosen for ballistic application. Additionally, the application of epoxy with the addition of grapheme nano palate (GNPs), carbon nano-tube CNTs, hexagonal boron nitride nanosheets (BNNS), and boron nitride nanotubes (BNNT) nanoparticles as the matrix for the composites reinforced with glass fiber fabric was described. The authors assessed the ballistic impact behavior and damage mechanisms of the glass fiber reinforced plastics (GFRP) modified with different nanoparticles (0.25 wt% GNP; 0.1 wt% CNT; 0.1 wt% CNT:0.1 wt% BNNS and 0.25 wt% GNP: 0.1 wt% BNNT [11]. Composite from aramid and epoxy presented good reinforcement-matrix homogeneity, with an overall fiber content of about 60% and possess high flexural strength for composite with 18 layers [44].

### 3.4. Compression and Hot Press Molding

Salman et al., study the properties of hybrid composite consist of plain woven kenaf and aramid fiber reinforced with PVB phenolic with different arrangement and thickness. This composite was fabricated by using hot press technique. Hybrid composite showed high impact compare with single fiber layer and meet the production of NIJ standard [36]. Rahman et al. [41] study on performance of E-glass/epoxy composites adding with amino-functionalized multi-walled carbon nanotubes (NH2-MWCNTs) to improve ballistic performance. Based on the experimental analysis, it can be reported that the addition of MWCNTs at 0.3 wt% loading increased the ballistic limit velocity by about 6% whereas higher loading of MWCNTs did not increase the ballistic limit. Previous study on carbon hybrid composite was used ultra-high molecular weight polyethylene (UHMwPE) for hard ballistic panels that can improve the back face signatures (BFS) as well as structural behavior, without affecting its ballistic limit performance. The presence of a stiffer carbon fiber composite layer on the UHMwPE hard ballistic panel on the outside had improved the bending rigidity of the panel [42].

### 3.5. Injection Molding

Injection molding is a process mainly consisted of three stages which are filling, packing/holding and cooling. Injection molding was a popular method for producing bullet proof system. Combination of three types of polymer materials which are thermoplastic polyurethanes (TPU), PP, and polycarbonate (PC) were injected into the pores of the re-entrant honeycomb structure by injection molding process to form the composite sandwich layer. Compare with this materials, PP has highest tensile strength followed by PC and TPU [43].

### 3.6. Extrusion

The extrusion process is composed of a series of physical, thermal and chemical changes occurring simultaneously or consecutively inside the extruder barrel. The characteristic of the product is related to the time that a particle spends in the extruder [60]. Previous researcher was studied on ammonium perchlorate (AP) as oxidizer, aluminum (Al) as metallic fuel, and TPU as binder for ballistic application. The polymer with additive and binder shows high thermal stability compare with pure polyurethane [61]. Next researcher reported the use of co-extruded tape technology to create all-PP composites with a large temperature processing window above 30 °C, and high-volume fraction of reinforcement above 90%. The large temperature processing window of these co-extruded tapes allows all-PP composite production over the range of this temperature processing window by providing enough thermal energy [62]. The output of the product depends mostly on the processing conditions, i.e., temperature and extruder speed. Extrusion is a typical process of manufacture. Blending polymer using melt blending or twin-screw extruder technology in manufacturing is an attractive method of producing high-performance compounds. Therefore, there have variety of processing method for producing body armor and ballistic from natural fiber. For comparison, fiber diameters used in soft body armors are several times smaller than that of human hair. The selection on fiber material is crucial to fit on the specific application.

**Table 4:** List of various manufacturing technique in ballistic application.

Material	Manufacturing Technique	Ref.
Non-woven kenaf fiber and Kevlar	Hand lay up	[31]
Aramid and woven kenaf fiber	Hand lay-up	[29]
Aramid, polyethylene and pineapple fiber	Hand lay-up	[30]
sisal fiber, polyaramide fiber, Epoxy	Hand lay-up	[32]
Woven kenaf, Kevlar hybrid yarn, epoxy	Hand lay-up	[33]
polyaramid/vinyl ester, Kevlar fiber	RTM	[34]
Woven fabric, unsaturated polyester resin	RTM	[35]
Plain woven kenaf, aramid and PVB phenolic	Hot Press	[36]
Single and yarns fiber (carbon, glass and para-aramid fiber), epoxy	Hand lay-up	[37]
Glass and graphite fiber with epoxy	Vacuum Infusion	[38]
Carbon, aramid, and epoxy	Vacuum Infusion	[39]
Kevlar and thermosetting resin	Hand lay-up	[40]
Graphene nanoplatelets, glass fiber and epoxy	Vacuum Infusion	[11]
E-glass and epoxy	Hot press	[41]
Polyethylene fiber and carbon fiber and epoxy	Hot press	[42]
thermoplastic polyurethanes, polypropylene, and polycarbonate	Injection molding	[43]
Aramid and epoxy	Vacuum Infusion	[44]
Ammonium perchlorate (AP) as oxidizer, aluminum (Al) as metallic fuel, and thermoplastic polyurethane	Extrusion	[61]
Polypropylene composite	Extrusion	[62]

**Table 5:** Advantages and limitations of polymer composite manufacturing techniques.

Manufacturing Technique	Advantages	Limitations
Hand lay-up	Simple principle to apply, higher fiber volume, and longer fibers size can be produced.	Product quality is not stable and depend on the skills and experience of the skilled worker.
Resin transfer molding	Provides better uniformity, controlled surface on both sides of panel, high fiber volume and low void contents.	More expensive tooling than compression molding.
Vacuum infusion	High consistency and repeatability.	Higher consumable costs with lower slower cycle times.
Compression and hot press molding	Lower cost tooling.	Not suitable for complex mold and slower processing time.
Injection molding	Fast production and highly efficient.	High tooling cost and longer set-up lead time.
Extrusion	Ability to produce complex cross section material, high volume production and consistent resin usage.	High initial cost for set-up.

## 4. Natural Fiber Treatment Techniques as Adhesion Promoters

Natural fiber when compared to the synthetic fiber will prevail as a growing importance of reinforcing substance. Extensive applications of plant driven natural fiber as reinforcement for polymers in various applications is not surprising as natural fiber usually referred to provide several advantages over synthetic fiber such as easy availability, eco-friendly, moderate modulus-weight ratio, high acoustic damping, low density, low manufacturing energy consumption, biodegradable, sustainable and low carbon footprint [9,63,64,65,66]. Furthermore, the major demand for new material of natural fiber is rising due to the further growth in economics of countries and the whole world at the same time. Despite listed major benefits that have been offered by natural fiber, there are still significant challenges impeding their advancement into the field of various structural applications, since major areas are limited to the interior and non-structural applications [67,68,69].

One of the major limitations of using natural fiber as polymer reinforcement is their hydrophilic behavior in nature which makes them incompatible with polymer matrices. Natural fibers are known to have hydrophilic characteristics due to the presence of large amounts of hydroxyl groups; which later results in the absorbed moisture content to be as high as 30 to 95% relative humidity in value [70]. This extremely hydrophilic nature behavior of natural fiber results in the difference in the polarity state that will lead to poor interaction and compatibility between natural fiber and hydrophobic polymer matrices, thus producing composites with low properties [71,72,73]. In addition, this highly polar cellulosic fiber provides the tendency of natural fiber to absorb moisture which affects the fiber-matrix interaction bonding that can lead to the failure of composites and subpar performance [74].

Low thermal stability is another problem that limits the use of natural fiber in reinforcement of polymer matrices. Thus, it can be deduced that the temperature at which natural fibers are exposed during composites processing is usually limited to 200 °C in order to avoid the degradation of natural fibers during thermal processing [75]. On this basis, as consequences, choices of polymers that can be used as the potential matrix for natural fiber reinforced composites have been surpassed. Other aspects of challenges include fiber/matrix adhesion, fire resistance, durability, manufacturing difficulty and variability in quality [76]. Moreover, composites reinforced with plant fibers are depending on the other aspects as example; fiber-matrix ratio, filler material, matrix properties, processing techniques and coupling agent [22].

Implementation of modification of natural fiber is crucial as a solution to overcome the shortcoming of materials. In general, modification techniques of natural fiber have been categorized into two types, which are physical and chemical treatment methods. Both physical and chemical modification of natural fibers are normally performed to alter the limitations of natural fiber reinforced polymer, usually to improve functional properties such as wettability, dimensional stability, thermoplasticity, therefore increase their bonding and adhesion with hydrophobic matrix. The physical treatment method applied for the natural fiber is an important aspect to purify, oxidize and ultimately activate the surface of the fiber. This treatment type of process results in structural and surface properties changes of the fiber.

### 4.1. Physical Treatment Methods of Natural Fibers

Several treatments techniques for natural fiber under physical treatment methods have been identified by numerous researchers, such as fibrillation, electric discharge (cold plasma, corona) and many more. This type of treatment change the surface and structure properties of the fibers without application of chemicals and improves the bonding between the polymer matrix and the reinforcement fiber-matrix thus increases the strength of the fabricated composites [26,77]. Table 6 depicts the well-known examples of the physical treatment methods of natural fiber available in the market.

The treatment with Corona has been used mainly to increase the surface energy by minimizing the limitations effect of moisture on properties of composites, which results in increasing the adhesion between the fibers and polymer matrix [85]. The longer the time taken for the fiber was being immersed in the corona treatment, the coarser the surface of the fiber would be. This coarser surface of the natural fiber improves the mechanical bonding between the fiber and matrix. Corona treatment approach in this study leads to the remarkable improvement in mechanical properties of hemp-reinforced polypropylene composites as high as 30% value of increment [86]. However, there is some difference findings in another study revealed by Koohestani et al. [87] which indicated that the performance of corona treatment on some fibers reduced as much as 20%, in this case, when poly(lactic acid) (PLA) as a matrix being applied with the fiber. Another study by Gassan et al. [86] mentioned that the application of corona treatment to modify the surface of jute fiber has increased the polarity of fibers but decreased the fiber strength, which results in downgrading composite strength. Based on the published literature, it can be summarized that the corona treatment only modifies the surface structure of the natural fibers with no definite findings to claim the retention of the fiber constituents through the fiber treatment. The resulting effect of the corona treatment on some part of research results in weak mechanical bonding.

Plasma treatment has induced the significant progress of natural fibers in which it alters the surface structure of natural fibers by decreasing the weakly attached layers in the fiber, which it can be elucidated through the new formation functional groups of free radicals, ions, and electrons. The substrate is bombarded with high energy particles travelling in the stream of plasma during the process of plasma treatment [88]. The plasma produced as the treatment of fiber had a positive impact on the surface roughness and chemistry, wettability of the substrate without the usage of any solvents or hazardous chemicals. Plasma treatment can be generated for the modification of surface structure of natural fiber through removal of weakly attached surface layers by abrasion and cleaning process, thereby creating new functional groups (functionalization and cross linking).

This type of physical treatment is usually carried out for both matrix and fiber. Nevertheless, there are no significant changes occurring on the emerging composite materials [89]. As eloquently stated by George et al. [85], partial areas of the treatment process is conducted in a molecular gas enclosure that contains proportion of molecules in a vacuum chamber, whilst the incorporation of matrix or fiber will contain electrons, radicals or ions in allowing the changes in surface structure. It has been shown earlier that the modified approach by plasma treatment to both sisal fibers and high-density polyethylene matrix has been conducted Ramamoorthy et al. [90]. As aforementioned, the use of plasma treatment has been investigated for the treatment of wood fibers and sisal fibers, by applying argon and air as the plasma feed glass [91]. The mechanical properties of the resulting wood fiber and sisal fibers reinforced PP composites through plasma treatment had been increased as compared with the untreated natural fibers. The tensile strength improved by as much as 16% for both wood fiber and sisal fibers reinforced PP composites. At the same time, tensile modulus was found to be significantly increased as much as 127% for wood fiber and 93% for sisal fiber respectively. This is a result of greater interfacial adhesion between fibers and matrix as a result of plasma treatment.

Besides low-pressure plasma treatment, the modification surface of natural fibers in terms of wettability and interfacial adhesion between matrix and fibers can be improved by using atmospheric air pressure plasma (AAPP) treatment, and therefore this process will remove non-cellulosic substances from the surface of these fibers. Low operating cost, shorter treatment time and greater flexibility as no vacuum system is needed could be achieved by using AAPP treatment. In order to overcome the disadvantageous effect generated by nitrogen and oxygen feed gases on lignocellulosic fibers, compressed air can be applied as an alternative gas.

Great interfacial adhesion can be achieved when the surface tension of the natural fibers is far greater than that of the matrix [92]. In other words, critical surface tension can be modified through AAPP treatment. As studied by Baltazar-y-Jimenez et al. [92], by increasing the treatment time on the abaca treated fibers, the critical surface tension had been increased. Furthermore, the longer treatment times of hemp and sisal fibers brings the crosslinking of the surface of hemp and sisal fibers, Moreover, there is another finding whom reported by Baltazar-y-Jimenez et al. [92] that the AAPP treatment on the properties of natural fiber reinforced cellulose acetate butyrate composites. The storage modulus of the short fiber reinforced composites had increased as high as 370% at 30 wt% of fiber loading fraction. This strongly indicates that AAPP treatment is able to increase the fiber-matrix interaction between the fibers and matrix. Moreover, in terms of the thermal properties, the mechanical glass transition temperature had been increased and the height of tan delta had displayed better fiber-matrix bonding and fiber-matrix distribution.

Superheated steam is usually conducted under normal atmospheric pressure as described by Li et al. [89]. A regular tap water had basically been used in this superheated steam treatment process. In general, the temperature of superheated steam was pre-set to 220 °C and allowed to reach a steady state. Natural fibers will then uniformly be poured on the aluminum foil tray with a dimension size of (10 × 12 × 1 cm^3^). Afterwards, it was subjected to the heating chamber of superheated steam for an oven for 1 h. Fiber was then removed immediately from the heating chamber, cooled in a desiccator, and ultimately kept in the sealed polyethylene bag to be used in analysis.

**Figure 7:**
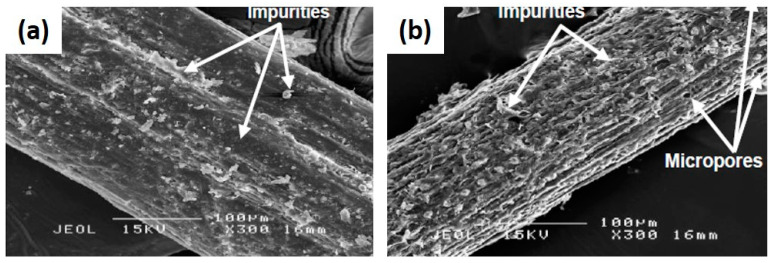
Superheated steam treatment method of application in (**a**) untreated (**b**) and treated OPMF.

The effectiveness of the modification of fiber through superheated steam had been seen in the experimental research carried out by Then et al. [82]. The degree of interfacial adhesion and contact between fiber and matrix could be examined through a study of surface morphology by SEM method. A relatively clean and rough surface of oil palm mesocarp fiber (OPMF) obtained from the treatment process in comparison to the untreated fibers is illustrated in Figure 7. The treatment was carried out by eliminating the impurities as well as non-cellulose substances. Similarly, Edeerozey et al. [93] in their study stated that during fiber treatment, the silica particle had been revealed after the partial removal of those impurities and non cellulose substances. It could be seen that there were small micropores on the surface of treated fiber, indicating some of the silica particles that were previously being embedded in the fiber had been removed. Thus further facilitating both mechanical interlocking and bonding reaction [94].

Furthermore, the increment in the thermal stability of biocomposites could be found with an improvement in the cellulose percentage of fibers as a result of superheated steam treatment. The resulting high cellulose percentage in treated fibers is crucial for the fabrication of biocomposites as it provides the strength to the biocomposites. Comparing the strength of cellulose with hemicellulose and lignin, cellulose is found to give higher strength than the other constituents after super-heated steam treatment, as shown in Table 7 [82].

Upon treatment of fiber, hemicellulose would be the first component to be eliminated due to the lower value of thermal and chemical resistance of hemicellulose as compared with cellulose and lignin [95]. This finding is supported with the other outcome from other researchers on the superheated steam-treated oil palm empty fruit bunch fiber by Bahrin et al. [96]. Generally, increasing in thermal stability of the corresponding biocomposites is depending upon the increasing percentage of cellulose in treated fibers. Additionally, lower moisture content (3.74%) of treated OPMF as compared with untreated OPMF (7.87%) can be explained as it contained high percentage of lignin but a minimal percentage of hemicellulose. Treatment of fiber minimizes the disadvantageous effect of moisture on properties of composites, consequently, adhesion between fibers and polymer matrix can be improved, which later upgrades the strength of composite.

Gamma radiation is another method of modification that provide feasible way to harden/toughen/strengthen the materials by subjecting to crosslinking, chain-scissions, decomposition and unsaturation within the polymeric chains [97]. Commonly, gamma ray irradiation is essentially being performed in the industrial process for polymeric material improvement. Gamma ray from Cobalt-60 (highly penetrating rays) for the treatment of fibers is considered as one of the developing technologies at the industrial scale. Back then during the seventies, radiation processing of polymeric raw materials was normally carried out with electron accelerators of medium or low energy of electrons, limited only for the surface treatment and a few millimeters of death.

The development of gamma ray correlates with the strong capacity of penetration of the irradiation was particularly adapted in the eighties, allowing the treatment of larger contact area and thickness of bigger products of the size of cupboard box (several centimeters) up to a pallet (1 m). Radiation processing of molded parts, especially involving packaging and of complete big bags or bobbins would be carried out Le Moigne et al. [98]. Today, there are wide variety types of research conducted using gamma ray irradiation, in the case of lignocellulosic substrates; preferably work is carried out in the dose range of 1 to 50 kGy. In addition to this, various researchers have preferably conducted using gamma ray irradiation for not more than 30 kGy due to the possibility of biopolymers chain scissions of the original properties of natural fibers that can degrade drastically. In fact, the resulting performance of biocomposites could be affected as well. The cost price for the serial industrial treatment will be lower than 1 $/kg for radiated material, while in the case of large delivered volumes, the price would be as lower as 0.3 $/kg that can be obtained Le Moigne et al. [98]. It should be noted that this outcome could lead to the economic feasibility of radiation processing of natural fiber. Referring to the irradiation conditions, the natural fiber structural changes occurred upon irradiation through cross-linking, intermolecular bonding and oxidation mechanisms. It is necessary to emphasize the strong impact on the structural and a microstructure property of natural fiber was basically derived from these phenomena of gamma radiation.

The promising outcome from the development of ballistic bulletproof vest had been revealed through significant mechanical properties results of tensile, impact and flexural tests of treated kenaf hybrid/X-ray composite [84]. The surface of kenaf fiber had been treated with sodium hydroxide together with an X-ray radiation and did improve the interfacial bonding adhesion between kenaf hybrid composite. From all of the samples tested, configuration sample from the combination of surface treated X-ray together with NaOH solution had been chosen as the optimum formulation with the aim of producing a specimen with hybrid composite properties for bulletproof vest application.

Based on Table 8, the results of mechanical properties for the configuration sample of kenaf/NaOH/X-ray treated composite had shown as the best formulation among other samples been tested. It was observed that the configuration of kenaf/NaOH/X-ray hybrid composite sample had resulted in moderate flexural modulus and tensile strain values as compared with the other types of samples, which include kenaf/NaOH treated, kenaf/Xray treated as well as untreated kenaf composites. This combination type of kenaf/NaOH/X-ray sample had been selected as the most appropriate formulation for bulletproof vest armor application. Materials that are having moderate strength properties which are not too soft, which in turns will make them as weak and not too hard, which make them be-come uncomfortable to be wear are desired for bulletproof vest application. The treated specimen had improved the interfacial bonding between the two distinct materials, therefore, had formed no delamination. On the other hand, the untreated specimen had displayed obvious delamination at the top part of the image. In addition to this, the ability of sample material to withstand high impact force up to 838 N and absorb significant impact energy up to 138 J showed that the design is qualified as a high velocity impact resistance for bulletproof and ballistic applications.

The effectiveness of the ballistic performances of the materials can be modified through improvement of surface coefficient of friction from a study of application of alkaline treatment together with a silane coupling agent for the modification of kenaf natural fiber surface with high density polyethylene (HDPE) for ballistic panel vest application (Akubue, P.C., Igbokwe, P.K., & Nwabanne, J.T. (2015). Production of kenaf fiber reinforced poly-ethylene composite for ballistic protection. IJSER, 6(8), 1–7). It is reported that the ballistic panel vest of kenaf/HDPE depicts the ballistic/penetration resistance with three different shots of calibers, which is shown in Table 9. Alkaline mercerization treatment of kenaf natural fibers resulted in strong absorption and dissipation of energy impact that were transmitted from the bullet to the ballistic vest, making the bullet to deform or being ”mushroom”. Afterwards, this could further be explained by the scenario that the bullet is basically caught in a web of strong fibers from the vest, which then resulting the bullet struck the body armor in the end. Excessive energy has been absorbed by each successive layer of material in ballistic vests. The perfect strong combination between treated kenaf natural fiber with HDPE as a matrix for vest works as a large area of ballistic vest in preventing the bullet from penetration. As a result, the dissipating force had end up caused blunt trauma or non-penetrating injuries to the internal organs.

Another strong aspect of findings from this study of ballistic panel vest of treated kenaf fiber/HDPE is its impact strength properties, which is depicted in Table 10. The high impact energy value of 774.4 J had contributed to the blunt trauma protection especially from any possible injury by the strong ballistic panel vest.

Another research study was made on bulletproof panel applications in order to investigate the impact of chemical solvents such as ethanol, acetone, methyl ethyl ketone together with a silane as coupling agent on the ramie woven fiber [99]. As the world is looking forward towards the safety of the environment as well as the need for improved performance and low cost, this bulletproof panel is suitable to be developed as a new material which is believed having lower economical cost than conventional bulletproof available in the market that were made mostly from Kevlar/aramid composite, steel-based material and ceramic plate. In addition, this bulletproof panel is considered having lighter in weight that could act as a potential alternative material for the military standard ballistic equipment.

Other characteristic and attractive features of bulletproof panel from treated ramie woven fiber, include the high velocity impact from the penetration of bulletproof testing. This silane treated ramie woven fiber contributes significantly to the resistance of penetration of high impact projectile through the sufficient breaking strength and toughness for bulletproof testing. With the help of silane treatment addition on the ramie woven fiber, full metal jacket projectile was stopped and trapped by the bulletproof panel. Besides, it is to be noted that the ramie treated with silane having higher tensile strength value (1219 MPa) than the ramie treated with ethanol (1143 MPa). Furthermore, it is also mentioned that the value of moisture content (%) properties of ramie treated with silane is 5.77%, which is lower than the value of ramie treated with ethanol (6.33%). As a consequence, ramie woven fiber treated with silane produced good quality fibers. In view of this, concluding remarks from this study indicated that the silane treated ramie woven natural fiber had the ability to be implemented as reinforcement materials for bulletproof composite panels for future military applications.

### 4.2. Chemical Treatment Methods of Natural Fibers

It is well established that the modification of reinforcing fibers can be obtained by chemical method as well. Modification of fiber by chemical means is important for the increment in the amount of amorphous cellulose at the expense of crystalline cellulose. Removal of hydrogen bonding in the network structure is an important modification to be expected in this process. Among various chemical treatments available, alkaline treatment or mercerization is one of the most frequent treatments used to reinforce thermoplastic and thermoset polymer composites. Basically, mercerization is a process of breaking down of the composites fiber bundle into smaller fibers known as fibrillation. The reduction of fiber diameter increases the aspect ratio that leads the rough surface topography resulted in enhance fiber-matrix interface adhesion and improve mechanical properties [100]. During this process, the unstable groups of hydroxyl will be disintegrated and reacted with water lead to elimination of reactive ionized molecules to produce alkaline oxide. It is expected that the surface roughness of cellulose fiber is improved and hydrophilic groups are removed [97] as Equation (1):

Fiber–OH + NaOH → Fiber–O–Na^+^ + H_2_O (1)

In this mercerization process, alkali cellulose is formed as a result of sodium hydroxide penetration into crystalline regions of parent cellulose (cellulose I). The formation of cellulose II, regenerated cellulose will take place after the process of washing out unreacted NaOH from fiber. Alkaline treatment brings two important effects on the fiber [89]. The first one is the structural surface of fiber turns into roughness, resulting in better mechanical interlocking. In addition to that, it can also increase the amount of possible reaction sites by increasing the amount of cellulose exposed on the surface of fiber [101]. Alkaline treatment successfully removed hemicellulose and lignin that are considered as the non-cellulosic component of fiber, consequently bringing lasting effect for producing a close-packed cellulose compound. Thus, the crystallinity of the fiber increases after forming the treatment process as the close-packed cellulose is linked to each other through hydrogen bonding [102]. In other words, the fiber surface become cleans and become more uniform due to elimination of micro-void, lead to enhancement of stress transfer capacity between ultimate cells [103].

**Figure 8:**
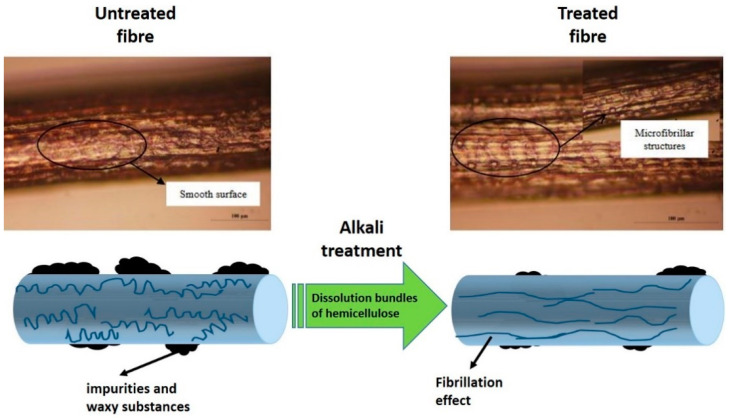
Schematic mechanism of the untreated and treated natural fiber using alkaline treatment.

Figure 8 shows the schematic mechanism of the untreated and treated natural fiber using alkaline treatment and effect of alkaline treatment under optical microscope for sugar palm fiber at 1% NaOH concentration for 1 h soaking time. As a result, the tensile strength (coupon testing) of treated sugar palm fiber was increased from 156.92 MPa to 332.28 MPa. Based on study by Nurazzi et al. (2019), all the mechanical properties (tensile, flexural, impact and compression) showed improvement after the alkaline treatment of sugar palm yarn fibers compared with the untreated sugar palm fiber hybrid composites. This was due to an improvement in compatibility and better adhesion between the sugar palm yarn fiber and glass fiber with the matrix [51]. In term of thermal stability study of the hybrid composites, the 50/50 wt% fiber ratio of the 40 wt% treated sugar palm yarn fiber showing good fiber interactions resulting in compatibility between fiber and matrix that reduces the damping factor of the composites and shows the highest glass transition temperature (T_g_) at 82.50 °C [104].

However, excess of alkali would lead to delignification of cellulose which able to damage or weaken the structure. Rodriguez et al. [105] indicate limitations on the extremely high concentration of alkali and/or too long time taken of the alkaline treatment with various concentrations. From all of the mentioned concentrations, it was stated that 5 to 6% alkaline treatment with treatment duration of 2 to 3 h is sufficient enough to remove the hemicellulose and lignin [106]. The significant effect of mercerization or alkaline treatment on flexural properties of unidirectional PP/flax composites is listed in Table 11. As expected, the value of flexural strength for the treated flax reinforced PP composite was higher than that of untreated flax reinforced PP composite. Based on study by Zin et al. [107], the study showed that the optimum alkaline treatment for banana fiber is 6% NaOH concentration with a 2-h immersion period, which resulted in 371 MPa tensile strength, 12.45 GPa tensile modulus and 3.96 MPa interfacial shear strength. The tensile strain increases with higher NaOH concentration. As the concentration increases beyond 6%, the mechanical properties of banana fiber deteriorate significantly.

The treatment of fiber with liquid ammonia had usually been carried out purposely for cotton. This type of treatment had been developed since the late 1960s as a substitute to mercerization. Liquid ammonia penetrates quickly the interior of cellulose fibers, as it is having low viscosity and surface tension, creating a complex compound in the end of the treatment process due to the rupture of hydrogen bonds. After the process of liquid ammonia treatment, the original crystal structure of cellulose I had changed to cellulose II and cellulose III. Therefore, at the following stage, cellulose III had changed to cellulose I again after hot water treatment [78]. Deconvolution and smoothing effect of the composites could be obtained based on lignocellulosic surfaces. Concurrently, cross section of fiber becomes round and lumens decrease [108].

**Figure 9:**
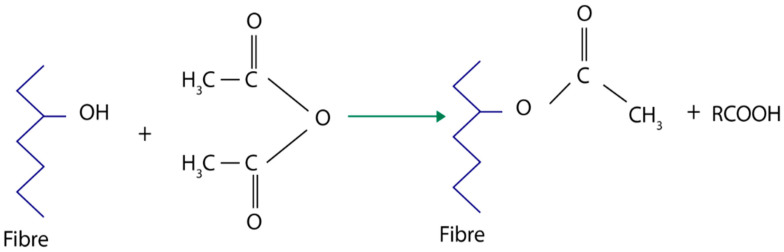
Reaction process of acetic anhydride with cellulosic fiber.

Chemical modification through esterification is usually conducted through typical esterification as well as etherification reactions of lignocelluloses hydroxyl groups. The reaction with organic acids or anhydrides is referred to as esterification. Huge types of esters are possible depending on the nature of organic acid (anhydrite) applied in the reaction. Acetylation was found to be the most popular esterification method which has been developed in commercial scale, starting first in the United States [109] followed by Russia [110]. The scale up of the process by several groups had been progressively done within Europe. The reaction process of acetic anhydride with fiber is revealed as Figure 9.

This process involves fiber plasticization by incorporating the functional group of acetyl. Acetyl acid (CH_3_OH) was used to react with hydrophilic hydroxyl groups of fibers and remove the existed moisture. Hydrophilic hydroxyl groups in lignocellulose had been substituted by hydrophobic radicals. This later then, the properties of fibers especially equilibrium moisture content (EMC) could be altered. Due to this process, modification had altered the fibers polarization and further improved the compatibility of fiber to non-polar matrix. The process of esterification provides rough surface tomography with lower number of voids. The esterification of plant cellulose fiber also helps to improve the hydrophobicity properties as well as the stress transfer properties at coalesce and structural features (impact, flexural and tensile) of laminates take place [97].

Another common chemical treatment is silane modification. Silane is synthetic compound that used as coupling agent to modify the surface of the fiber. The composition of silane forms a chemical link between fiber and matrix surface through siloxane bridge [103]. The basic mechanism of silane modification involves the reaction of silane agent, alkoxysilane with the surface of prosperous hydroxyl that attach with polymers at the an-other ends. The modification comprises of several stages including hydrolysis, condensation and bond formation. During the hydrolysis, silanol form in the presence of moisture and hydrolysable alkoxy groups. The condensation process then occurs as one of the silanol reacts the cellulose hydroxyl group (Si-O-cellulose) whilst the other end reacts with matrix functional groups. This reaction provides molecular continuity at the interface of the composites as well as provides hydrocarbon chain that restrain s the fiber swelling into the matrix. The formation of hydrocarbon chains also provides an active covalent bond be-tween fiber and matrix which lead to better adhesion. This modification resulted improvement in fiber-matrix adhesion and stabilize the composite properties [103]. Silane also able to mixed with water to produce silanol and further reacted with hydroxyl group of cellulose, hemicellulose and lignin through the linkage of ether thus eliminate the water content [97].

Another type of chemical treatment method for natural fiber is acrylation and maleic anhydride treatment. The treatment of fiber through acrylation reaction at hydroxyl groups of fiber depicts as Equation (2):

Fiber–OH + CH_2_=CH–COO–Na → Fiber–O–CH_2_–CH_2_–COOH (2)

Subsequent reaction at the interface is expected at the curing time of composites with the occurrence of peroxide decomposition. Higher temperature is preferable for the decomposition of peroxides to take place. The equation can be shown as Equations (3) and (4):

RO–OR → 2RO^−^ (3)

RO^−^ + Fiber–H → R–OH + Cellulose^−^ (4)

**Figure 10:**
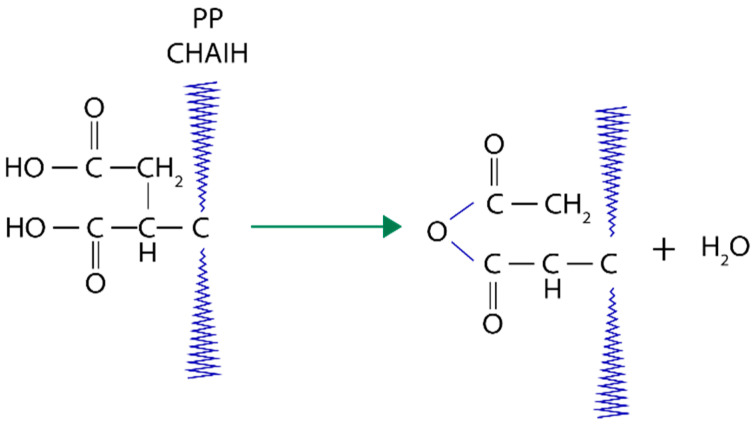
First method of treatment of cellulose fibers with MAPP copolymer.

**Figure 11:**
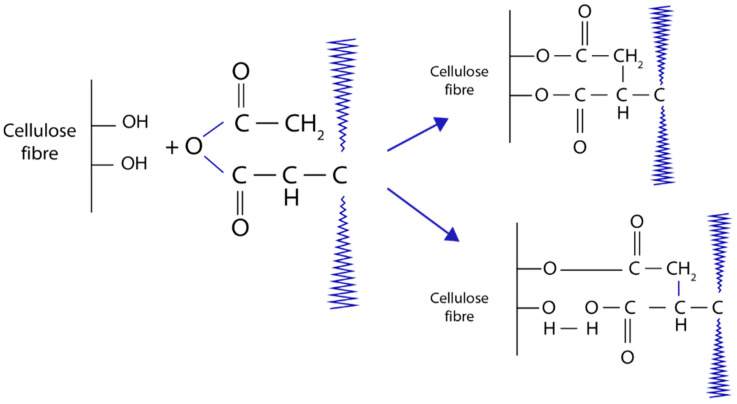
Second method of treatment of cellulose fibers with MAPP copolymer.

Covalent bonds across the surface as a result from the treatment of cellulose fibers with hot polypropylene maleic anhydride (MAPP) copolymers (Figure 10). Basically, from this process, there are two methods of obtaining biocomposites from natural fibers and polymers. In the first technique, pre-treated fibers with maleated polymer are reinforced with the selected polymer matrix. While for the second method (Figure 11), polymer, fiber, and maleic anhydride with the combination of peroxide initiator in one step processing are actively extruded and followed with molding or injection to obtain a final desired composite.

Surface structure of natural fiber could undergo chemical treatment of fiber by graft copolymerization. Several properties such as grafting proportion, grafting efficiency, and grafting efficiency are affecting the degree of compatibility of cellulose fibers with a matrix polymer. It is then stated by Thomas [111] that the grafting parameters are influenced by the concentration and type of the initiator, by the monomer to be grafted and the reaction conditions. As a result of the graft copolymerization process, the resulting copolymer has the unique properties characteristics of both fibrous cellulose and grafted polymer. The monomer molecule is more accessible to the active center of cellulose in water than in an organic solvent. The grafting reaction could preferably proceed between cellulose and acrylonitrile without the presence of lignin. Further effect on the mechanical performance of oil palm fiber using several types of surface modifications is depicted in Table 12 [112].

Despite several benefits and advantages of physical and chemical treatment, however, it is worth noting that there are several limitations emanating from physical and chemical treatments of fibers as shown in Table 13.

## 5. Natural Fiber Reinforced Polymer Composites for Bullet Proof and Ballistic Applications

Rising demand for natural fiber composites increased rapidly due to low density, high specific strength, abundance in nature, good thermal properties, cost-effectiveness, and most important is biodegradable [45,50,113,114]. In recent years, the development of natural fibers as reinforcement in polymer matrix for defense application and ballistic-resistant composites was get interested among researchers. The ballistic composite commonly used for helmets, body armor, vests, and for shield components on military vehicles. The most common types of composite ballistic materials used are Kevlar, Twaron, high-molecular weight polyethylene (HMWPE) (Dyneema) and ultra-high-molecular weight polyethylene, (UHMWPE) (Spectra). The study regarding on incorporation of natural fibers in ballistic material application was listed in Table 14.

### 5.1. Natural Fiber Composites Reinforced with Thermosetting Polymer

**Figure 12:**
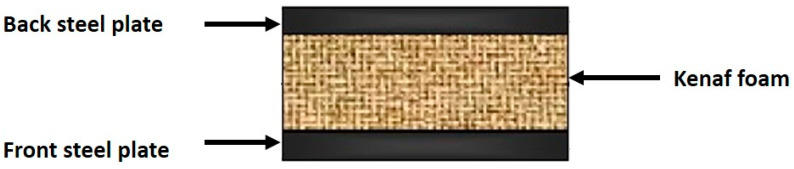
Cross sectional of sandwich armor plate with kenaf foam.

Numerous studies have been conducted on the properties and characteristics of kenaf fiber reinforced polymer composites for ballistic purposes. Zainol Abidin et al. [115] investigated the suitability of kenaf fiber and polyurethane (PU) foam sandwich with steel plates as shown in Figure 12. The effect of different percentages of kenaf fiber (10, 20, and 30) and thickness of the foam inside the armor plate (15, 30, and 45 mm) on the impact resistance and behavior of sandwich panel was investigated. PU foam with 20% kenaf and thickness of 45 mm was found to have a lower depth of indentation compared to the other samples. They also concluded that this type of samples has high resistance impact behavior against the ballistic impact of the bullet. Therefore, the PU foam strengthening with kenaf fiber increased the impact resistance properties for manufactured composites compared with the neat PP.

Azmi et al. [116] manufactured a bulletproof vest from woven kenaf and X-ray films using different layers of composite configuration, with epoxy resin as a matrix. In this research, epoxy-based hybrid composites were produced using the hand lay-up method and their flexural and high velocity impact ware tested. There are 4 types of panels from different configuration, that consist of 7 layers of woven kenaf and X-ray films. The result showed that X-ray films produced better in both properties compared to woven kenaf and hybrid composite. They concluded that the interfacial bonding between two different materials was the major problems that lead to the composite delamination. Additional study by utilizing chemically treated woven kenaf and treated X-ray film by punctured their surface was conducted. Total eleven types of composite at different configuration layers were produced and tested for their tensile and flexural properties. It was found that both properties were improved in the composite made from both treated materials. Composite with the configuration of three layers’ surface treated X-ray films sandwiched between two layers of treated woven kenaf, was selected to be the base design for the specimen subjected to impact test. Even the composite made up from full woven kenaf presented highest tensile and flexural properties, with some consideration in term of comfort and high flexibility, hybrid treated materials was selected. The good interfacial bond between treated woven kenaf and films was noticed as the major contribution, as well as high in impact energy absorption.

**Table 14:** List of reported study on utilization of natural fiber reinforced polymer composites for ballistic applications.

**Thermoset Polymers**					
**Fiber Types**	**Matrix Type**	**Ballistic Limit (m/s)**	**Energy Absorption (J)**	**Application**	**Ref.**
Kenaf fiber (10, 20, 30%)	Polyurethane	-	57–120	Ballistic protection materials	[115]
Jute fabric (10, 20, 30 vol%)	Polyester	-	200–260	Ballistic material	[117]
Kenaf fabric and X-ray films	Epoxy	-	111–143	Bulletproof vest	[116,118]
Non-woven kenaf and Kevlar	Epoxy	165–255	121–324	Ballistic laminate composites applications	[31]
Woven coir and Kevlar	Epoxy	-	-	Body armors	[119]
Woven kenaf and Kevlar (30, 50, 70%)	Epoxy	-	39–148	Ballistic laminate composites	[33]
Jute fiber (10, 20 and 30 vol%)	Epoxy	-	-	Components of ballistic armors	[120]
Fique fiber	Epoxy	-	-	Ballistic armor	[121]
Curaua fiber (10, 20, 30%)	Polyester	-	-	Personal ballistic protection	[122]
Sisal fiber (30%)	Epoxy	-	106	Ballistic armor	[123]
Bamboo fiber (30%)	Epoxy	-	-	Portable armor for personal protection	[124]
Coir fiber (10, 20, 30%)	Epoxy	-	-	Personal ballistic protection	[125]
Ramie fiber and Kevlar (15, 25 mm)	Polyester	623–837	1362–3185	Anti-Ballistic board for body armor	[126]
Pineapple leaf fiber (PALF) (30 vol%)	Epoxy	-	-	Bulletproof vest	[30]
**Thermoplastic Polymers**					
**Fiber Types**	**Matrix Type**	**Ballistic Limit (m/s)**	**Energy Absorption (J)**	**Application**	**Ref.**
Flax, hemp and jute fabric (46 vol%)	Polypropylene	-	-	Ballistic material	[13]
Kenaf fiber	Polyethylene	-	774	Ballistic panel vest	[127]
Kenaf fabric and aramid	Polyvinyl butyral	477–621	-	Combat helmet	[36]
Kenaf fabric and aramid	Polyvinyl butyral	417–496	-	Combat helmet	[128]
Chonta palm wood (10, 20, 25, and 30%)	High density polyethylene	-	41–53	Biocomposite armors	[129]

**Figure 13:**
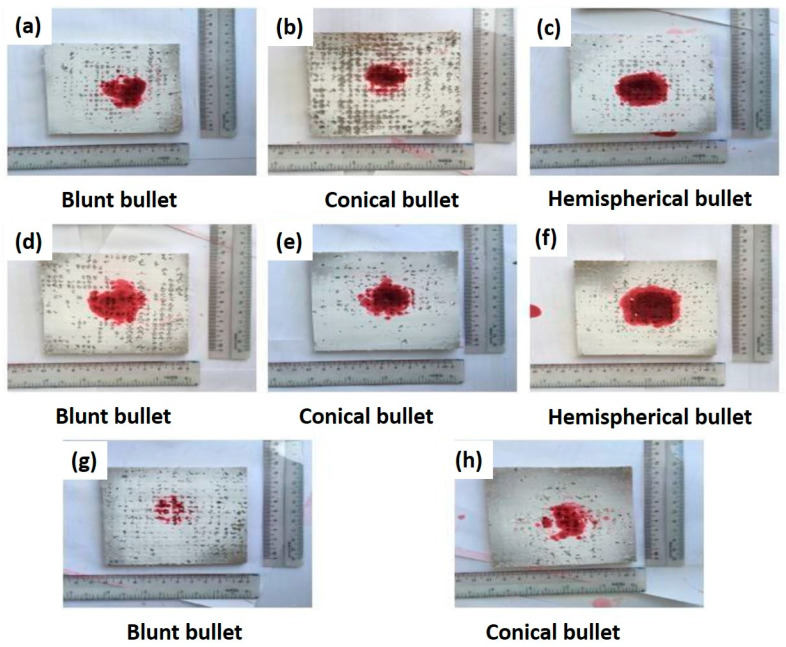
Dye penetrant test shows damage area of; (**a**–**c**) 30 bar pressure setting; (**d**–**f**) 40 bar pressure setting; and (**g**,**h**) 50 bar pressure setting. (Adapted with copyright permission from Azmi et al. [130]).

The experiment on several pressure settings of gas gun (20, 30, 40, and 50 bar) and different steel projectiles used (blunt, hemispherical and conical) in the high velocity impact of composites performance was conducted by same researchers, Azmi et al. [130]. In this study, 10 layer of treated hybrid composite; 4 layers of X-ray films were sandwiched between 3 layers of kenaf fiber on top and at the bottom, was produced by hand lay-up and using epoxy resin. From the results, they found that this multi-layer treated hybrid composite was suitable for ballistic materials because it can withstand a projectile movement of up to 240 m/s and able to absorb up to 135 J of impact energy. Among the types of projectiles used, hemispheric projectile has the highest penetrative potential compared to blunt and conical projectiles. The hemispheric projectile was able to completely penetrate the specimens at a pressure of 50 bar. From the analysis of damaged specimens, it was observed that most of the bullets penetrated into woven kenaf that behaves like the ceramic front of a MAS, and bounced back or remained trapped in the X-ray film layer at the center of the composite that act as an impact resistance material. They also conducted a penetrant test on the impacted specimens to observe the damage area and their progression. The showed that the hemispheric projectile had left a wide and deep area of damage from strong penetrative intensity (Figure 13c,f), while the conical projectile has significant damage, with small region compared to hemispheric projectile (Figure 13b,e,h). Additionally, visual inspection also shows that the hemispheric projectile produces the highest impact, resulting in a deeper indentation of the laminated hybrid composites.

**Figure 14:**
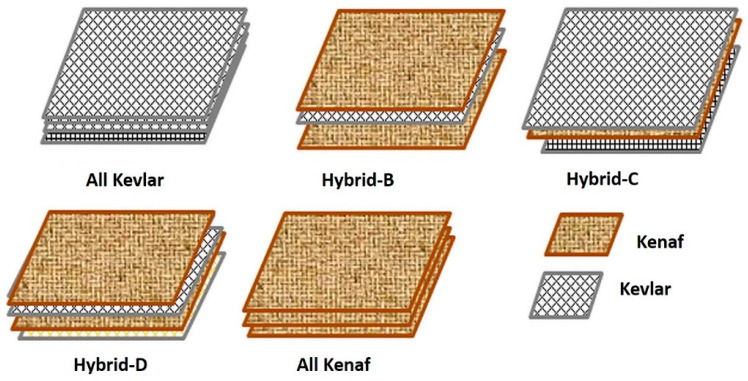
Hybrid of kenaf-Kevlar with different layering sequences. (Adapted with copyright permission from Yahaya et al. [31]).

In ballistic laminate composites applications, the use non-woven kenaf of reinforced epoxy composites was established. Yahaya et al. [31] investigated of hybrid of non-woven kenaf mat and Kevlar with the effect of layering sequences, as well as the hybridization effects as shown in Figure 14. Quasi-static penetration resistance and high velocity impact test was conducted on all samples. It was stated that the penetration energy of hybrid composites increased in the hybrid kenaf-Kevlar composites compared non-hybrid composites. Hybrid composite with Kevlar as the outer layers showed stronger penetration force and energy absorption, contributed by the great coefficient of friction of Kevlar, which resists the development of a complete shear plug and prolongs the load–displacement curve, with the assist of kenaf mat that having different stiffness and friction coefficient. However, a ballistic limit and energy absorption during ballistic impact was found lower in the hybrid composite. Compared to hybrid composite, Hybrid B absorbs higher ballistic impact energy than Hybrid C and Hybrid D, suggesting that more ballistic impact energy is absorbed by hybrid composite with kenaf at the outer layer. Hybridization of kenaf/Kevlar created variation the in successive rear part layers’ restraining factors, thus lead to the reduction of the contact time with a projectile on the previous layer [131].

In addition, Yahaya et al. [132] also conducted experiments on the effect of kenaf contents and fiber orientation (woven, 0°/90° cross ply unidirectional, and non-woven mat) on tensile and flexural properties of kenaf/Kevlar hybrid reinforced epoxy composites. The results showed that the hybrid composite tensile behavior enhanced by 14% when using unidirectional kenaf yarn structure, compared to woven kenaf composite. They stated that such increment in tensile properties was contributed by the fracture mechanism of kenaf yarn in the matrix that involves fiber/matrix debonding, fiber pull-out, stress distribution due to fiber fracture and multiple fibers. Furthermore, kenaf yarn has higher breaking strain and modulus of the individual fibers that others [133]. For flexural properties, woven kenaf composite was found has highest flexural strength, as a result of higher fabric density, fiber structure and the location of resin-rich areas. However, in term of fiber content, composites with high in kenaf content show similar trend in all fiber orientation; flexural and tensile strength decreased with the increased of kenaf loading. This are due to the fiber’s failure to support the stresses transmitted from the polymer matrix, and low interfacial bonding partly creates gaps between the material of the fiber and matrix, resulting in poor structure.

Yahaya et al. [29] investigated woven kenaf-Kevlar epoxy composites with the effect of woven kenaf hybridization, layering sequences, thickness and areal density on the ballistic limit velocity (V50) properties and energy absorption. In this study, two types of samples were prepared, Type A and B. Type A comprise of Kevlar/epoxy that consists of 9, 15 and 21 layers, while type B consist of kenaf-Kevlar/epoxy that consists of Kevlar hybrid layers with additional two layers of woven kenaf. Compared to non-hybrid Kevlar composites, woven kenaf-Kevlar hybridization has resulted in lower specific energy absorption of the composites as compared with non-hybrid Kevlar composites. However, the additional kenaf layers in hybrid composites contributed to an increase in the thickness and density of composites, thus increasing the absorption of energy and ballistic velocity, due to larger travel distance that increase of surface for energy dissipation. The hybrid composites deteriorated in the impacted surface, kenaf-Kevlar interface and rear surface by a mixture of fiber shear, delamination and fiber fracture.

**Figure 15:**
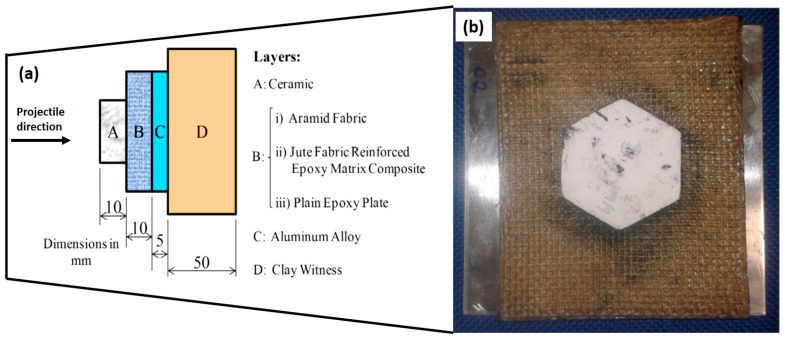
(**a**) Schematic diagram of the multi-layered armor system (MAS) that consist of jute fabric reinforced with epoxy, and (**b**) real composite sample. (Adapted with copyright permission from Da Luz et al. [134]).

Da Luz et al. [134] has carried out the ballistic impact characteristics of a MAS with 30 vol% jute fabric reinforced epoxy composite and plain epoxy plate as shown in Figure 15. The purpose of this study is to identify the impact properties of the composite when the second layer in the MAS system was substituted with jute reinforced epoxy composite. A depth penetration, impact velocity, residual velocity and internally dissipated energy in individually ballistic composite were evaluated according to NIJ 0101.06 standard, by using 7.62 mm ammunition. As noted by the higher energy dissipated by jute epoxy composite, the jute epoxy composite had better impact performance than plain epoxy and Kevlar. When subjected to projectile impact, the front ceramic tile suffered by the impact force. The disruption area caused by the explosion of fragments of the jute epoxy composite showed the splitting of the jute fiber into thinner fibrils, which is a feature of its mechanical rupture, indicated absorption of more energy. Interestingly, weight and cost analysis concluded that by considering comparable ballistic efficiency and negligible weight disparity, the significantly lower costs associated with the environmental advantages of a natural fiber favor the replacement of aramid and plain epoxy jute fiber composite in a MAS.

**Figure 16:**
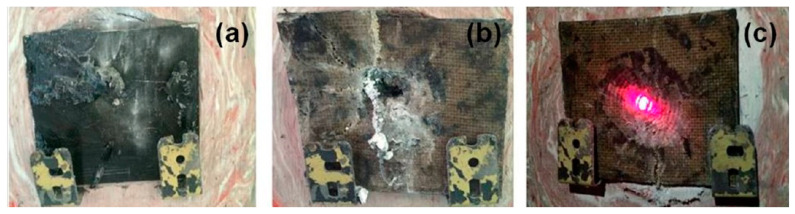
Images of jute fabric at (**a**) 10, (**b**) 20, and (**c**) 30 vol% as second layer in the multi-layered amour system (MAS) polyester composite. (Adapted with copyright permission from Monteiro et al. [117]).

Monteiro et al. [117] analyzed the effect of jute fabric content namely 10, 20, and 30 vol% on composite integrity and waves impedances in the multi-layered amour system (MAS). The commercial materials used in the second layer in the MAS system is aramid fiber such as Kevlar and Twaron, but in this study the layer was replaced with jute fabric. The jute fabric was reinforced with polyester resin and pressed into thickness of 10 mm, and this layer was interlacing between ceramic tile and aluminum alloy sheet as a third layer in the MAS system. Using the 7.62 mm bullet, ballistic tests were carried out on the composite specimens according to NIJ Standard class III, where the bullet velocity, shock waves impedances, and failure images were measured. They found that composites manufactured using woven jute reinforced with polyester and used as a second layer had successful meet the ballistic standard requirement, where the depth of indentation must be smaller than 44 mm. The presence of jute polyester composite prevented the third layer that consists of aluminum alloy to perforate, and created small depth indention in the clay witness. Among three types of composites, 30 vol% of jute fabric had the less fragmented, where the material still intact in the MAS composite, compared to the 10 and 20 vol% of jute fabric, that showed completed fragmented, and partially fragmented, respectively (Figure 16). The mechanism of mechanical instruction and fragment attraction by Van de Walls forces and static charges from the jute fabric that contributed in high energy absorption was found to be the main factor of better strength. Interestingly, they also found that the depth of indention in a clay witness simulating a human body protected with a MAS at velocity of 7.62 mm bullet was same within the statistical precision in the jute fabric and Kevlar uses as a MAS second layer. The lowest shock impendence value that represents the depth of the indentation in the clay witness, indicated high energy absorbed, was found in the composite contain 10 vol% of jute fabric. However, due to the complete fragmented after the bullet test, this type of composite was not suitable to use in MAS second layer for multiple shots personal protection.

### 5.2. Natural Fiber Composites Reinforced with Thermoplastic Polymer

For the thermoplastic matrix, earlier studies on natural fibers in 2007 for ballistic application was conducted by Wambua et al. [13]. They explored and compared the ballistic properties of natural fiber reinforced polypropylene composites (NPP composites) with NPP-backed steel plate composites and NPP-coated steel plate composites as facing and backing materials. The natural fibers used were jute, flax and hemp. In his research, Wambua et al. had discovered that flax reinforced polypropylene composites manufactured by hot compression molding show the highest energy absorption effect with a V50 of 312 m/s when compared to hemp and jute composite. The ballistic limit increased with the introduction of steel plates. As 0.8 mm thick mild steel was used as facing and backing to the composites, the V50 showed large increments (109%).

**Figure 17:**
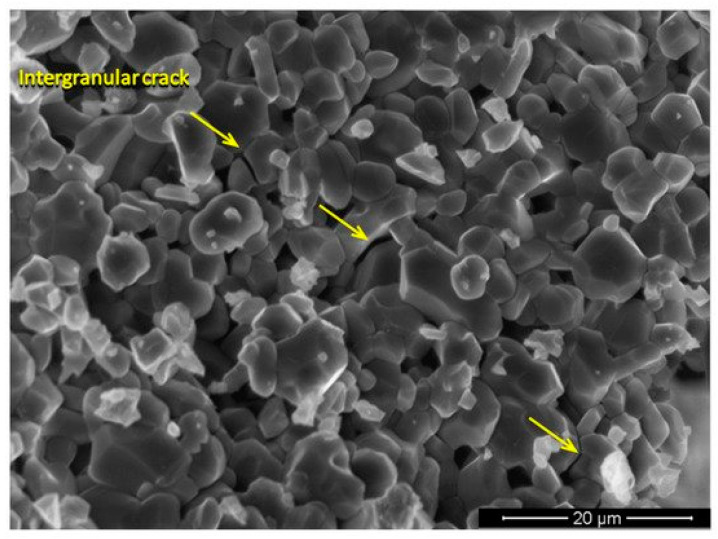
SEM image of ceramic intergranular fragmentation after ballistic test. (Adapted with copyright permission from Da Luz et al. [134]).

**Figure 18:**
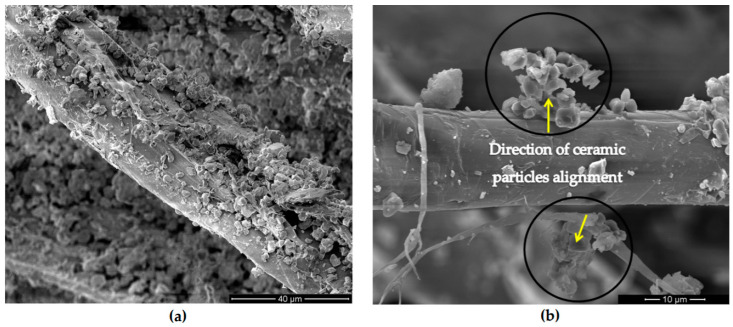
SEM images of the fracture surface of the PALF composite after the ballistic test: (**a**) PALF impregnated with ceramic aggregates; (**b**) alignment of ceramic aggregates on PALF surface. (Adapted with copyright permission from Da Luz et al. [134]).

Whereas, when the 1.5 mm steel plate was used to face the composites, the V50 improved by around 50%. The analysis in the variation of V50 increased in areal density and thickness, modes of failure, and variation in energy absorption power. The main modes of loss observed were delamination, fiber breakup, shear cut-out, and localized bulging. The ceramic intergranular fragmentation and fracture surface of PALF fiber reinforced polymer composites under ballistic impact are shown in Figure 17 and Figure 18, respectively. They concluded that the dominant mode of failure relied on the composite panel’s characteristics and could change during its failure.

Salman et al. [84] investigated the suitability of using plain woven kenaf reinforced with Polyvinyl Butyral (PVB) phenolic to replace Kevlar fabric in the production of ballistic helmets. Several configuration layers and stacking sequence of the hybrid laminates and helmets were studied. They have shown that the hybridization of kenaf/Kevlar PVB composites have a higher potential for absorbing impact energy but a lower energy absorption was found in the composite with alternate layer of woven kenaf than the placing woven kenaf layers together. They mentioned that by placing woven kenaf together and Kevlar layers separately is more efficient in the laminated hybrids because of the degree of delamination was higher. The different flexibility and deflection in both kenaf and Kevlar affected the magnitude of friction forces, leading to a decrease in the impact energy absorption mechanisms [135]. The average high-speed effect test findings reveal that approximately 30% of the volume fraction of both kenaf and Kevlar fibers is more effective for the energy absorbed contributed by the fact that 30% of the fibers has stronger interfacial surface properties, contributing to an improvement in surface area for energy dissipation. The findings of the impacted helmet and trauma deformations suggest that the existence of the woven kenaf and PVB film has a favorable influence on the posterior deformation of the helmet shell.

Haro et al. [129] used chonta palm wood micro particles as reinforcement in composites of high-density polyethylene (HDPE) for ballistic application. To assess its mechanical and ballistic performance, quasi-static and dynamic tests were performed on composites and indicated that the mechanical properties are improved by reinforcement with particles of chonta palm wood. Therefore, the incorporation of chonta palm wood microparticles as reinforcement into a polymer matrix such as HDPE is a promising method of creating biocomposites with enhanced capacity to endure complex loading of impacts and absorb energy from impacts.

## 6. Hybrid Natural Fiber Reinforced Polymer Composites for Bullet Proof and Ballistic Applications

To date, researchers have been aware of the significance of natural fiber applications. The fact that, the natural fibers can impart better ballistic properties and promoting green composites concept has made it the materials of choice. The reasons mainly rely on its ability to provide lightweight properties with reasonable strength and if engineered effectively, such composites can be a good product as body protective armor which it can give more comfort to the wearer in addition to being environmental friendly [5,30]. The body protective armor or bulletproof equipment includes bulletproof vests and helmets are known to be made from high strength synthetic fibers such as aramid, Kevlar, ultra-high molecular weight polyethylene (UHMWPE), nylon, glass [136]. Natural fibers has been the focus of the researchers and developer as the alternative to synthetic fibers in composites for bulletproof and ballistic applications and usually applied in the form of woven or knitted fabric or in some cases, encapsulated or embedded in composites materials [136].

Development of the technology in manufacturing high performance fibers have enabled the production of advance composites for body protection application. This includes the hybridization of synthetic fiber with natural fibers, natural fibers with other natural fibers and the application of nanomaterials as second filler. In the formation of bulletproof and ballistic application, the hybrid system can be defined as a system that consists of layers of materials bonded together can serve specific purpose of preventing projectile penetration. Designing ballistic materials from hybrid composites are now becoming more popular due to its promising results and achievement. The application of natural fibers were reported can increase the performance of the synthetic fibers [137]. However, the hybrid system needs more than one types of materials to achieve enhance in properties. The increment of material used causes increase in its weight. Therefore, studies need to be conducted to develop less dense multi-layered armor system for better bulletproof and ballistic properties [125].

Hybrid combination can be comprised of (1) two or more reinforcing phases embedded in single continuous matrix, (2) single reinforcing embedded in two or more matrices and (3) two or more reinforcement incorporated in multiple matrices. The advantages of hybrid composites attributed to the superiority of one type of constituent could surpass the limitation of other constituents. Hybrid for multi-layered of ballistic system usually made up of two or more high performance fibers [5,138].

The properties of hybrid materials are generally depended on the aspect ratio of fiber, properties of individual fiber, orientation of fiber, length of individual fiber, adhesion between fiber and matrix and stacking sequence of both fibers. The properties of the hybrid composites of two elements can be estimated through the rule of mixtures as shown in the Equations (5) and (6):

P_H_ = P_1_V_1_ + P_2_V_2_ (5)

R V_1_ + V_2_ = 1 (6)

where, P_H_ is the property analyzed, P1 the corresponding property of the first element and P2 the corresponding property of the second element, V_1_ and V_2_ are the volume fraction of first and second element respectively [139].

**Figure 19:**
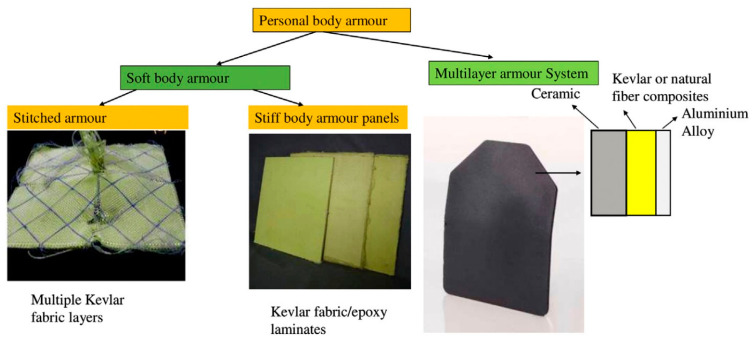
Different types and fabrication techniques for body armor.

Meanwhile, the ballistic and bulletproof performance of the hybrid composites were analyze based on its ability to absorb energy, velocity limitation, and the depth of indentation of the natural fibers hybrid composites. This performance is needed in the production of efficient body armor. Figure 19 showed different types and fabrication techniques of body armors. According to Roy et al. [140] and Naveen et al. [138], personal armor could be classified into soft armor and hard armor. Soft body armor then can be classified into stitched and stiff armor. Stiff armor contain multiple layer of fabrics up to 50 layers whilst stiff armor panel were fabricated using simple hand lay-up method made by Kevlar fabrics reinforced in the polymer matrix [141].

Meanwhile, the multilayer armor system was made with different combination for example ceramics/composites, ceramic/metals, composites/metals and ceramics/composites/metals. The combination of ceramic/composites/metal has been widely applied in defense sector. Ceramics are well known for its high ballistic properties and applied as striking face to improve effectiveness of multilayer armor. The second layer consist of composite materials used to absorb and dissipate the kinetic energy of ballistic threats or projectiles. The third layer comprise of metallic layer with function of stopping the impact energy carried by projectile. The three layers then were joint with polyurethane adhesive [56,138].

The importance features of fiber reinforced polymer hybrid composites for high velocity impact and ballistic application includes high specific strength and stiffness, impact resistance, crack resistance and low density. Noted that, the requirement for ballistic composites were differ to those that structural laminated composites. Those features include moderate fiber/matrix adhesion, higher fiber loading, moderate fiber impregnation and voids. The shape and size of the projectiles also play the important roles in energy absorption, ballistic limit and life of the body armor [138].

**Figure 20:**
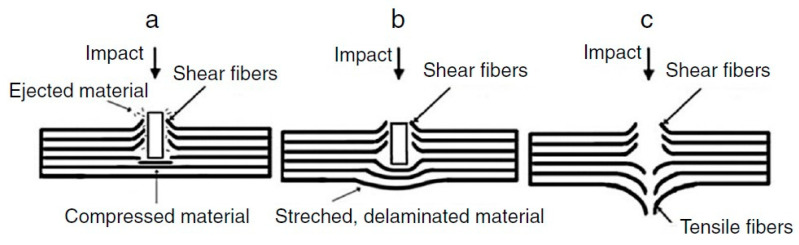
Ballistic failure mechanism proposed after the impact: (**a**,**b**) partial penetration and (**c**) complete perforation. (Adapted with copyright permission from Silva et al. [142]).

As reported from Naveen et al., [138], during the ballistic impact, the top layer of the panel exhibited shear failure whilst the bottom layer failed through tensile mode failure. Therefore, the hybridizing of different materials by sequencing the different laminas takes the advantage of individual constituents thus, improved the ballistic performance of the composite panels. The schematic mechanism of ballistic failure as shown in Figure 19 explained the effect of impact of a projectile, from partial penetration to complete perforation. Partial penetration consists of two stages. It starts with impact that lead to fiber breaking by shear followed by Figure 20a the kinetic energy of the projectile is absorbed and lead to speed decrement due to fiber stretching, bulging and delamination; Figure 20b complete perforation associated with fiber breaking which also lead to decrement of projectile speed due to energy absorption and lastly, Figure 20c plastic deformation indicate ballistic failure controlled predominantly by delamination and tensile stress at break of the reinforcing fibers.

To date, many researchers have reported the utilization of natural fiber in hybrid polymer composites. There are many forms of hybrid composites using natural fiber as one of the constituents, including such as natural fiber/synthetic fiber hybrid composites, natural fiber/natural fiber hybrid composite and natural fiber hybrid nanocomposites. The main aim of these hybrid composites is to replace/reduce the application of synthetic fibers at the same time, to develop advance material for ballistic and bulletproof applications.

### 6.1. Natural Fiber Reinforced Synthetic Fiber Hybrid Composites

Synthetic man-made fibers used for bulletproof and ballistic applications due to its heat resistance and extremely strong properties with exceptional strength-to-weight ratios. This materials was used to produce lightweight and flexible body armor that provide high level of protection and applied as the replacement of the conventional body armor made from metals, such as steel because they were heavy and often ineffective [136]. To date, the applications of e-glass, aramid fiber, poly-aramid fiber (e.g., Kevlar, Twaron and Technora), ultra-high molecular weight polyethylene (UHMWPE) or known as high performance polyethylene (HPPE) fibers and (e.g., Spectra and Dyneema), polybenzobis-oxazole, PBO (e.g., Zylon) and polypyridobisimi-dazole, PIPD (e.g.,M5) have been widely reported to be used as main material for ballistic and bulletproof protection armor due to its superior properties [30,136].

Owing to environmental problem, the replacement of natural fiber composites for synthetic fiber reinforced plastics has grown significantly due to lower cost and improved sustainability, which includes both advantages and disadvantages [137]. Currently, hybridization of natural/synthetic fibers as reinforcement in hybrid composites has shown promising effect on the improvement of ballistic and bulletproof properties. The application of natural fiber to reduce the application of synthetic fibers will enhance the environmental performance compare to those that pure synthetic fiber reinforced polymer composites [128].

Aramid fibers is known as high strength fabrics owing to its high strength per unit. Several studies have investigated the hybridization of aramid fibers with natural fibers to impart better properties as well as lower the cost of manufacturing. Salman and Leman [128] in their study reported the application of aramid fibers with plain woven kenaf fabric reinforced polyvinyl butyrate (PVB) composites. PVB is thermoplastic used as interlayers that widely used for laminated safety glass. The combination of hybrid aramid/woven kenaf reinforced in PVB were successfully able to withstand fragmentation, and 9 mm ammunition ballistic protection up to threat third level II-A and confirmed NIJ standard.

Naveen et al. [141] in their work, has produce multi-layered composites using Kevlar and *Cocos nucifera* sheath reinforced epoxy composites. Layers of hybrid composites with different sequence was fabricated via hand lay-up method. The hybridization between Kevlar and Cocos nucifera sheath was found enhanced the ballistic performance compared to Kevlar/epoxy composite. Two stages gas gun was set up together with high speed camera to find impact velocity and residual velocity of the projectile. High energy absorption was reported for hybrid epoxy composites attributed to lower cellulose and high lignin content of Cocos nucifera sheath that absorb and dissipate the kinetic energy of the projectile away from the impact zone.

Among all other natural fibers, the one extracted from pineapple leaf *(Ananas comosus)* also denoted as PALF is known as the strongest natural fibers and have been extensively studied as reinforcement in polymer composites. The ultimate stress of PALF can reach over 1.6 GPa and elastic modulus above 80 GPa which can help on imparting enhancement on the strength and stiffness of any polymer matrix [56]. Da Luz et al. [30] in their study manage to fabricate the hard armor consist of two distinct layer of ceramic and PALF fiber reinforced epoxy. The incorporation of 30 vol% of PALF fiber in the composites system showed better performance compared to ceramic layer reinforced ultra-high-molecular-weight polyethylene (Dyneema) with lower back-face signature (BFS) depth (26.6 mm) which meet the NIJ standard for ballistic protection against a rifle.

**Figure 21:**
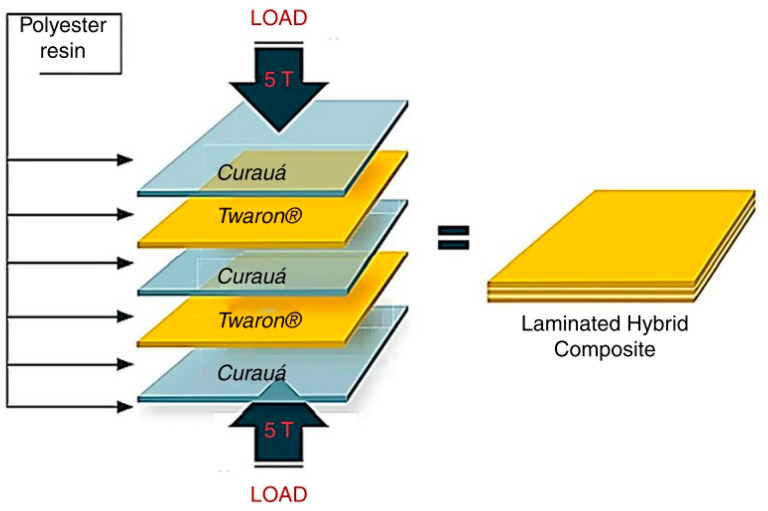
The schematic representation of caraua reinforced aramid fabric hybrid composites.

The effect of natural hybrid composites as exposed to ultraviolet (UV) radiation also has been investigate. The exposure of composites when used outdoor might change its behavior. Therefore, da Silva et al. [142] in their study has investigate the ballistic behavior of hybrid composites reinforced with caraua and aramid fabric subjected to ultraviolet radiation. The schematic representation of the caraua/aramid hybrid composites preparation was depicted in Figure 21. Though, the composites produced from caraua fiber is considered one of the strongest, the results have shown that after the exposure by UV radiation for 300 h and 600 h has affect its ballistic performance. The UV radiation leads to delamination on the interface of plies, chain scission on caraua fibers and increase crosslinking of the polyester resin. The absorbed energy from the impact of a 9 mm ammunition were reported decrease for 14% after exposed to UV radiation.

Despite of focusing only on the quality of hybrid composites on producing ballistic and bulletproof materials, the manufacturing cost and weight also taken into account. Azmi et al. [136] had considered the tensile and flexural characterization of new lighter and cheaper hybrid composites to replace materials in bulletproof vest. In this study, kenaf fiber was treated with NaOH and waste of X-Ray films reinforced in epoxy resin and fabricated via hand lay-up method. One of the configurations of hybrid composites consist of both treated materials showed excellent tensile and flexural strength. This configuration was used to be the based design for the specimen subjected to impact test. The impact performance of that design is qualified as high velocity impact resistant materials due to its ability to withstand impact force and absorb significant impact energy.

### 6.2. Natural Fiber Reinforced Hybrid Composites

The utilization of natural fibers in the laminated hybrid composites to completely replacing the synthetic fibers is already a promising line of investigation [10,143,144,145,146]. Despite of the effectiveness of synthetic fibers to provide superior ballistic performance, the effect of its application towards environment set the drawback of the materials. Therefore, the sole utilization of natural fibers in the composite panels for ballistic and bulletproof performance to completely replace the function of synthetic fibers were widely investigated. In terms of microstructure, yarn structure, fabric structure, etc., natural fibers are different compared to synthetic fibers which impart different energy absorption properties. Besides, different natural fibers possess different mechanical properties depending on their structure during natural growth. Therefore, good composites design can combine the advantages of various natural fibers to improve the overall strength of the composites materials [147]. However, very limited studies have been done for natural hybrid composites solely for ballistic and bulletproof applications.

**Figure 22:**
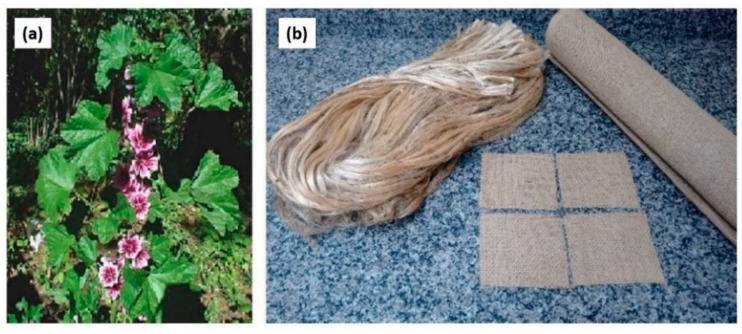
(**a**) Mallow plant; (**b**) mallow fiber, hybrid and pure fabric used to be incorporated in epoxy composites. (Adapted with copyright permission from Nascimento et al. [148]).

The study on the effect of natural fibers hybrid composites has been reported by Nascimento et al. [148] using mallow and jute natural fabrics reinforced epoxy for multi-layered armor. Through the study done, 100% mallow fabric, 70/30 and 50/50 of mallow fabric and jute fabric, respectively can be considered as suitable material for use in ballistic shield as all these materials meet the requirement of standard NIJ 0101.06 as shown Figure 22. The analysis of variance also confirmed that the similarity of mallow and jute fabric in applications was confirmed and found comparable to the Kevlar. Besides, the application of hybrid mallow/jute fabric reinforced epoxy able to reduce the total cost of multi-layered armor system (MAS) with weight decrement by 4% compared to those that aramid fabric.

Meanwhile, Shen et al. [147] in their study reported the effect of low velocity impact on hybrid natural fiber reinforced composites. The combination of jute and ramie fiber were applied to be reinforced with epoxy resin with various stacking sequence was found able to resist penetration of low velocity impact with optimum ratio of ramie and jute at 55:45. Despite of using thermoset or thermoplastic as the matrix, many research has been done on shear thickening fluid (STF) on its performance of excellent ballistic and bulletproof properties. The STF is a dense suspension whose viscosity increase rapidly with increase shear rate and this materials has widely been studied for puncture resistant products and bulletproof applications [149,150]. Its ability to assist synthetic fibers especially Kevlar by enhancing their ballistic penetration strength and impact energy absorption have been widely reported [151,152,153,154,155].

STF materials also can be derived from natural materials. Corn-starch colloidal suspension is one of the best examples of natural STF that possess advantages such as extraordinary dissipation under impact or high shear force. Cho et al. [150] in their study have utilized the application of natural STF, cornstarch suspension with Korean traditional long fiber paper, Hanji to observed their effect on bulletproof performance. Through the evaluation, it was found that both Hanji and STF influenced the bullet penetration by two factors which are momentum and stress propagation. The stress momentum increased with increase Hanji layers and thickness of cornstarch. The finding of this work would be beneficial for developing cost effective and favorable bulletproof plates to enhance mobility of armors for the future.

### 6.3. Natural Fiber Reinforced Hybrid Nanocomposites

In order to utilize the function of natural fibers in polymer matrix, the hybridization of nanoscale reinforcement incorporated in the fiber reinforced polymer composites has been used particularly as the middle layer. Many researchers have investigated the effect of adding various nanofillers (graphene, carbon nanotube, nanocley and nanocellulose) ranging 0.01 to 5 wt% on the mechanical properties of fiber reinforced polymer nanocomposites.

To date, graphene-based materials have replaced the usage of aramid fabric based body armor. Lee et al. [156] has reported that the multilayer graphene has ten times greater specific penetration energy than that macroscopic steel. In order to take that as advantage, Naveen et al., [157] has investigate the effect graphene nanoplatelets (GnPs) to modify the epoxy resin and reinforced with Kevlar/Cocos nucifera sheath composites. However, the results showed the decrement in energy absorption and ballistic limit due to improvement in interfacial interaction between fiber and GnPs. This enhancement is inappropriate to absorb and dissipate the kinetic energy of the projectile.

In contrary, the addition of only 0.5 vol% graphene oxide (GO) incorporated in epoxy resin reinforced 30 vol% ramie fabric showed increment in ballistic absorption energy. Graphene oxide has known as one of the graphene based nanomaterials that synthesized from graphite through oxidation process [158]. Peirera et al. [159] found that the addition of GO in epoxy matrix reveals superior ballistic properties with presence of 30 vol% ramie fabric. The energy absorption of ramie fabric/0.5 vol% GO epoxy nanocomposites possess 23.4% higher compared to Kevlar alone. Therefore, the author claimed that the novel ramie fabric reinforced GO incorporated epoxy nanocomposites as a promising material for the second layer in a ceramic front multi-layered ballistic armor for personal protection.

Despite of using natural fiber reinforced with nanomaterials for advance ballistic properties, the application of biomaterial like nanocellulose also has been reported. Shear thickening fluid (STF) was known for its superior performance for ballistic and bulletproof applications. Wang et al. [149] in their study utilized the function of nanocellulose (CNF) to modify the properties of nanosilica STF and mix with Kevlar fabrics. The application of CNF was reported enhanced the steady state and dynamic aspect of the STF. With only 0.2% of CNF, the impact resistance of the composites was enhanced with almost no tear cross-section reported on the fracture surface.

## 7. Conclusions and Future Perspectives

Natural fibers or also well-known as plant cellulosic fibers are emerging substituents used in composite materials in vast fields. The natural fibers could provide significant contribution to national GDP of agro-based economies due to its availability, cheap, lightweight, biodegradable, high specific strength and modulus to weight ratio and less hazards to human health. In this manner, many material scientists and engineers conducted numerous research to integrate the NFRPCs with the current personal protective armors technologies recently. Based on numerous literature surveys, NFRPCs have shown a significant better ballistic resistance and energy absorption. For instance, basalt fibers have shown promising results in terms of higher ballistic limit velocity for NIJ level II type of ballistic applications. Apart from that, the energy absorption and ballistic limit of NFRPCs are almost matches with high performance Kevlar and aramid fabric composites. In this light, the abundance of natural fibers would provide this field with a cost-effective solution to ever-growing demand in global protective armor sector. These natural-based alternatives would aid the armor technology more accessible and affordable for combat personnel in conjunction to reduce the loss of human life. From this review literature, it also established that most of the natural fiber based hard body armors were tested for only one shot as in accordance with NIJ level III standards that the armor panel should not fail for six shots. In this point of view, extensive research is required to improve the ballistic performance of natural fiber based multilayer armors in order to commercialize the armor panels. Moreover, the middle composite layer was made with non-biodegradable polymeric composites. Thus, future research is suggested to apply natural fiber towards creating green armors by vary the weaving design of the natural fiber fabric. At the end, performance of natural based armor panels can be enhanced by adding the nanocellulose and thickening fluid as well as utilizing rubber parts in the armor structure system.

Yet there are numerous of review manuscripts available on the biocomposites, they are focused only on characterization, manufacturing, processing and other applications, but none of the reviewers have reviewed the mechanical performance specifically for bullet proof body armor structures. Here, we have attempted to present the recent progress in NFRPCs in modern armor applications with a detailed analysis of the abovementioned properties. Yet, the authors feel that there is presently a great need for a detailed review or research to be carried out in the application of NFRPCs in the field of defense technology for the fabrication of biocomposites armor.

## Figures and Tables

**Table 1:** List of standards on different armor type.

Armor Type	Test Round	Test Bullet	Bullet Mass	Armor Test Velocity	Hit Per Panel at 0° Angle	Maximum Back Face Signature	Hits Per Panel at 30° and 45°
IIA	1	9 mm, FMJ RN	8.0 (124gr)	373 m/s (1224 ft/s)	4	44 mm (1.73 in)	2
	2	40, S&W FMJ	11.7 (180gr)	352 m/s (1155 ft/s)	4	44 mm (1.73 in)	2
II	1	9 mm, FMJ RN	8.0 (124gr)	398 m/s (1306 ft/s)	4	44 mm (1.73 in)	2
	2	357, Magnum, JSP	10.2 (158gr)	436 m/s (1430 ft/s)	4	44 mm (1.73 in)	2
IIIA	1	357 SIG, FMJ FN	8.1 (125gr)	448 m/s (1470 ft/s)	4	44 mm (1.73 in)	2
	2	44, Magnum, SJHP	15.6 (240gr)	436 m/s (1430 ft/s)	4	44 mm (1.73 in)	2
III	1	7.62 mm, NATO FMJ	9.6 (148gr)	847 m/s (2780 ft/s)	6	44 mm (1.73 in)	0
IV	1	30 Caliber M2 AP	10.8 (166gr)	878 m/s (2880 ft/s)	1 to 6	44 mm (1.73 in)	0

**Table 2:** Physical and mechanical performance of natural fiber vs. synthetic fiber.

Fibers	Density (g/cm^3^)	Tensile Strength (MPa)	Elongation at Break (%)	Tensile Modulus (GPa)
Sugar Palm	1.292	156.96	7.98	4.96
Bagasse	1.5	290	-	17
Bamboo	1.25	140–230	-	11–17
Flax	0.6–1.1	345–1035	2.7–3.2	27.6
Hemp	1.48	690	1.6–4	70
Jute	1.3	393–773	1.5–1.8	26.5
Kenaf	1.45	215.4	1.6	53
Sisal	1.5	511–535	2.0–2.5	9.4–22
Ramie	1.5	560	2.5–3.8	24.5
Pineapple	0.8–1.6	400–627	14.5	1.44
Coir	1.2	138.7	30	4–6
E-Glass	2.5	2000–3500	0.5	70
S-Glass	2.5	4570	2.8	86
Aramid	1.4	3000–3150	3.3–3.7	63.0–67.0
Kevlar	1.44	3000	2.5–3.7	60

**Table 3:** Types of hybrid natural fiber/synthetic fiber reinforced polymer matrix use in ballistic application.

Hybrid Natural Fiber/Synthetic Fiber	Polymer Matrix	Remarks	Ref.
Woven kenaf and Kevlar	Epoxy	Using amine hardener	[29]
Pineapple leaf, aramid, polyethylene	Epoxy	Using triethylene tetramine (TETA) hardener	[30]
Non-woven kenaf and Kevlar	Epoxy	The resin was cured using joint amine type (905–3S)	[31]
Sisal fiber and polyaramid fibers	Epoxy	Sisal fibers was cutting to 3.5 mm in length. Then drying sunlight for 3 to 5 h eliminated moisture	[32]
Woven kenaf, Kevlar hybrid yarn	Epoxy	Kenaf fiber at 75.08 tex	[33]
Polyaramid, Kevlar	Vinyl ester	Kevlar fabric was cut in 300 × 300 mm^2^ pieces	[34]
Woven fabric	Unsaturated polyester Resin	Using 50% fiber volume	[35]
Plain Woven Kenaf, aramid	PVB phenolic	The stack of polymer composite consists of 19 layer	[36]
Single and yarns fiber (carbon, glass and para-aramid fiber)	Epoxy	Consists of 1,5 and 10 layer of polymer composite	[37]
Woven glass and graphite fiber	Epoxy	Fiber volume fraction for all types was 55%	[38]
carbon-aramid	Epoxy	Using different layer for each sample	[39]
Kevlar	Thermosetting resin	Average fiber weight fraction of 75% for each sample	[40]
Graphene nanoplatelets, glass fiber	Araldite epoxy resin	addition of graphene platelets (GNPs), carbon nanotubes (CNTs), combined hybrid hexagonal boron nitride nanosheets (BNNS)/CNT, and combined boron nitride nanotubes (BNNTs)/GNPs nanoparticles	[11]
E-glass fiber	Epoxy	Single fiber diameter of 14e16 mm was used. E-glass fibers were sized using epoxy silanes of max. 0.4% by weight	[41]
Polyethylene fiber and carbon fiber	Polyurethane and epoxy	Forms 12 layer of hybrid composite	[42]
-	Thermoplastic polyurethanes, polypropylene and polycarbonate	Forms a sandwich composite layer for bulletproof system	[43]
Aramid and Kevlar	Epoxy	Using plain Kevlar	[44]

**Table 6:** Physical treatment for the modification of natural fibers.

Method	Description	Ref.
Corona Treatment	▪ Named as air plasma ▪ Apply low temperature corona discharge plasma to convey changes in the properties of the surface of fiber ▪ Use of oxygen containing species ▪ Increases surface fiber roughness ▪ Improves wettability, polarity of the fibers and adhesion of plastic surface	[78,79]
Plasma Treatment	▪ Modify the surface of the fiber ▪ Reducing the weakly attached layers in the fiber ▪ Improve the surface fiber roughness ▪ Similar to corona treatment but performing using a vacuum chamber maintained at an appropriate pressure and gas composition ▪ Imparting hydrophobicity to the fiber surfaces, thus increasing the interfacial adhesion between the fiber and matrix	[80,81]
Superheated Steam	▪ Hydrothermal treatment of fiber ▪ Resulting in removing the hemicellulose part of fiber which is known to be the most thermally unstable and hydrophilic component in fiber ▪ Improving the fiber-matrix compatibility interaction	[82,83]
Gamma- ray irradiation	▪ Application of the high frequency or high energy of electromagnetic irradiation ▪ Radiation induced reactions in the macromolecules of cellulose are activated through the rapid localization of absorbed energy within the molecules to radicals ▪ Cellulose having carbon, oxygen and hydrogen atoms and has practically similar possibility of being ionized to be involved in chemical reactions (cross-linking and chain scission)	[81,84]

**Table 7:** Chemical compositions of untreated and treated OPMF.

Chemical Composition of Fibers (%)	Untreated OPMF	Super-Heated Stem OPMF
Cellulose	32.22 ± 1.54	42.54 ± 0.84
Hemicellulose	31.62 ± 0.46	18.73 ± 0.87
Lignin	23.89 ± 1.12	28.26 ± 0.68
Moisture	7.87 ± 0.70	3.74 ± 0.45
Ash	4.40 ± 0.60	6.73 ± 0.32

**Table 8:** Mechanical properties value of untreated and treated kenaf hybrid composites.

Configuration Types of Sample	Tensile Strength (MPa)	Tensile Modulus (GPa)	Tensile Strain (%)	Flexural Modulus (GPa)	Flexural Stress (MPa)
Kenaf/NaOH treated	470.3 ± 39.21	24.9	4.07	8.83 ± 0.05	90.59
Kenaf/NaOH/X-ray treated	396.9 ± 40.68	26.6	2.89	6.24 ± 0.01	32.08
Kenaf/X-ray treated	592.4 ± 42.08	26.6	5.20	1.21 ± 0.01	34.89
Untreated kenaf	269.6 ± 40.11	15.8	1.85	3.21 ± 0.66	15.15

**Table 9:** Ballistic test results of kenaf/HDPE panel vest.

Test No.	Caliber (mm)	Test Range		Penetration Depth/Level (mm)	Penetration Resistance
		Standing Distance (m)	Nozzle Distance (m)		
1	9	2.2	1.2	6.5	Yes
2	9	3.2	2.2	5.5	Yes
3	9	4.2	3.2	4.3	Yes

**Table 10:** Impact test results of kenaf/HDPE panel vest for ballistic application.

Ballistic Panel Type	Projectile (mm)	Mass of Projectile (g)	Impact Velocity (m/s)	Cavity Depth (mm)	Impact Energy (J)
Treated Kenaf/HDPE	9	8	440	24.05	774.4

**Table 11:** Flexural strength of untreated and treated flax reinforced PP composites.

Types of Fiber	Flexural Strength (MPa)
Untreated flax	77
Treated flax	115

**Table 12:** Mechanical performance of untreated and treated oil palm fibers.

Types of Treatment	Tensile Strength (MPa)	Young’s Modulus (MPa)	Elongation at Break (%)
Untreated	248	6700	14
Mercerised	224	5000	16
Acetylated	143	2000	28
Acrylated	275	11,100	26

**Table 13:** Summary of limitations for several physical and chemical treatment.

Corona	Plasma	Alkaline	Acetylation	Silane
- Differential properties of treated fiber as one of the fibers is exposed to electrode surface treatment - Emerging composite retains the properties of composites - Fiber treatment can result in etching effect	- Research on alternatives gases to replace oxygen are limited - Improvement in mechanical properties of the emerging composite is only visible when fiber length is 6 mm.	- Differential or non-uniformity in mechanical properties of emerging composite - Adaptation of LiOH and KOH to replace the existing NaOH is complicated - Disposal of waste/treated alkaline is costly and unsustainable - Treated fibers, sometimes, compact poorly with the matrix	- Acetic acid and acetic anhydride may sometimes not reactive with fibers - volume acid required is high adding up to the treatment cost - Inconsistencies in the mechanical properties of the evolving composite - Weight of treated fiber is reportedly high in some experimental results	- Excessive volume of silane is required which may constitute environmental challenges - Silane is often mixed with Alkaline for effective reinforcement thereby aggravating disposal of used chemicals - Flexural and impact strength of most emerging composite is insignificant until the alkane is mixed with silane

## Data Availability

Not applicable.
